# Soybean Root System Architecture Trait Study through Genotypic, Phenotypic, and Shape-Based Clusters

**DOI:** 10.34133/2020/1925495

**Published:** 2020-06-09

**Authors:** Kevin G. Falk, Talukder Zaki Jubery, Jamie A. O'Rourke, Arti Singh, Soumik Sarkar, Baskar Ganapathysubramanian, Asheesh K. Singh

**Affiliations:** ^1^Department of Agronomy, Iowa State University, Ames, Iowa, USA; ^2^Department of Mechanical Engineering, Iowa State University, Ames, Iowa, USA; ^3^USDA-Agricultural Research Service, Corn Insects and Crop Genetics Research Unit, Ames, Iowa, USA

## Abstract

We report a root system architecture (RSA) traits examination of a larger scale soybean accession set to study trait genetic diversity. Suffering from the limitation of scale, scope, and susceptibility to measurement variation, RSA traits are tedious to phenotype. Combining 35,448 SNPs with an imaging phenotyping platform, 292 accessions (replications = 14) were studied for RSA traits to decipher the genetic diversity. Based on literature search for root shape and morphology parameters, we used an ideotype-based approach to develop informative root (iRoot) categories using root traits. The RSA traits displayed genetic variability for root shape, length, number, mass, and angle. Soybean accessions clustered into eight genotype- and phenotype-based clusters and displayed similarity. Genotype-based clusters correlated with geographical origins. SNP profiles indicated that much of US origin genotypes lack genetic diversity for RSA traits, while diverse accession could infuse useful genetic variation for these traits. Shape-based clusters were created by integrating convolution neural net and Fourier transformation methods, enabling trait cataloging for breeding and research applications. The combination of genetic and phenotypic analyses in conjunction with machine learning and mathematical models provides opportunities for targeted root trait breeding efforts to maximize the beneficial genetic diversity for future genetic gains.

## 1. Introduction

Root system architecture (RSA) is essential for water and nutrient acquisition, microbe interaction, nutrient storage, and structural anchorage and impacts grain yield [1, 2]. Crop breeding programs including soybean rarely utilize RSA as selection criteria; therefore, RSA traits have developed indirectly in crop species [3]. Researchers are cognizant of the genetic and phenotypic complexity that is inherent at the organismal level and promote standardization in terminology and removal of redundancies for the measurement of every conceivable trait [4–6]. However, RSA studies have been hindered by trait, measurement, and environment complexity. The plethora of root traits identified through different studies and software further complicate the identification of opportunities to select the most informative and relevant suite of traits [5, 7–11]. A recent focus on the investigation of root trait methodologies has significantly advanced trait measurement capability and capacity [4, 12–17]. Continual efforts are needed to utilize genomics and phenomics tools to study the RSA trait variation for application in crop breeding and research programs [18].

Various systems have been introduced to study RSA traits including methods that focus on the controlled environment (lab bench, growth chamber, and greenhouse) and in the field environment [4, 13, 16, 17]. Controlled environments provide the ease of use, speed, and scalability required for crop breeding programs. Field environment methods can provide higher immediate applicability despite being more resource intensive and with lower trait heritability [4]. Researchers have attempted to gain insight through a balanced approach utilizing higher throughput systems with advanced technology together with field-based validation leveraging the advantages of both artificial and field-based methods while reducing their drawbacks [9, 19–21]. This insight will ensure a comprehensive understanding of RSA traits, their genome to phenome relationship, and trait selection targets for cultivar development. However, due to higher trait heritability and ease of scale, controlled environment experiments serve as a strong foundation for future RSA trait studies.

In each crop species, one of the first steps in utilizing traits for practical breeding outcomes starts with the exploration of its genetic diversity [11, 22, 23]. Limited information is available in soybean [*Glycine max* L. (Merr.)] for RSA traits, although some QTL studies with a limited number of genotypes have been published [24–26]. Soybean is an interesting crop for these studies due to a severe genetic bottleneck reported in cultivated varieties [27]. Despite the narrow genetic diversity of soybean cultivars in the USA, nonroot trait-focused studies have reported the value of germplasm banks being well equipped with large and useful genetic diversity [28–32]. Therefore, an initial scan to catalogue the diversity of root phenotypes within soybean genetic diversity is needed.

Morphology parameters are useful to classify roots into different types and to correlate root type to environmental advantages such as nutrient acquisition and drought or flood tolerance. For example, crop species with deep rooting systems have been correlated to adaptation in drought prone environments [1, 33, 34] while those with shallow, fibrous root systems have been shown to have efficient phosphorus uptake in phosphorus-deficient soils [35–42]. The “steep, deep, and cheap” root type in maize has been promoted for efficient and effective water and nitrogen acquisition [43]. A highly competitive root with fast-growing characteristics and efficient root placement, including deep roots, to chase moisture through the soil profile is most suitable for water deficit crop growing environments [1, 34, 44, 45]. Soybean taproots that elongate faster from germination also have been shown to burrow deeper into the soil profile, have increased root densities at depth, and are better able access to water in drought situations [46, 47]. A current dilemma is that optimum root architecture is based on the assumption that deep roots need to be complemented with shallow lateral roots to efficiently forage for soil immobile nutrients [36, 48, 49]. This dichotomy creates a need to further explore the elusive optimum root architecture. The initial step should be the compilation of reported root shape categories available in the literature (see Materials and Methods; iRoot categories), which can be accomplished through the combination of genetic and phenotypic information. For example, single-nucleotide polymorphisms (SNPs) can help determine genetic variability and create genotype-based clusters. Similarly, genetic diversity and RSA can be studied on a trait by trait or overall trait basis using phenotypic information in conjunction with principal component analysis (PCA), and hierarchical and k-means statistical methodologies allowing further insights to be drawn using these relationships [50].

The overall objective of this research was to catalogue soybean root trait diversity in controlled environment conditions and to investigate the correlation between genotype, phenotype- and nonroot phenotype/country of origin-based descriptors. This study was carried out using 292 diverse soybean accessions from the USDA core collection primarily in maturity groups II and III together with a subset of the Soybean Nested Association Mapping (SoyNAM) parents. These accessions were studied in controlled environment conditions and phenotyped with an imaging platform at 6 (6 d), 9 (9 d), and 12 (12 d) days after germination. Genotype-based clusters (GBC) were created using SNP data generated by 50K Illumina chip [51] in which genotypes were separated using hierarchical cluster analysis. Eight phenotype-based clusters (PBC) were created based on hierarchical clustering of thirteen root traits derived from the experimental study. Root shape-based clusters were created using averaged, smoothed, normalized, and compressed (high-level features) root shape outline data generated by Elliptic Fourier Transformation (EFT) and a Convolutional Neural Network (CNN). We created informative root (iRoot) categories based on characteristic root types described in the literature such as drought tolerant or nutrient foraging as a method looking beyond individual root traits to capture the essence of differing root shapes and characteristics. Our results indicate that soybean accessions for RSA traits are genetically diverse and cluster-specific trends and differentiations were evident. The US accessions showed limited genetic diversity, suggesting it could benefit from the infusion of useful RSA trait diversity in breeding programs. The approach present in this paper is applicable to other crops for RSA-focused breeding and research applications.

## 2. Materials and Methods

### 2.1. Experimental and Technical Design

We developed a mobile, low-cost, and high-resolution root phenotyping system composed of an imaging platform to establish a seamless end-to-end pipeline previously described in Falk et al. [52]. The platform includes obtaining root samples through image-based trait processing and extraction of biologically relevant time series data on root growth and development for phenomics, genomics, and plant breeding applications. The seedling growth system component allowed for easy removal from the growth chamber and nondestructive imaging at multiple time points (6, 9, and 12 days) after germination. The imaging platform component consisted of a Canon T5i digital SLR camera (lens: EF-S 18-55 mm f/3.5-5.6 IS II) (Canon USA, Inc., Melville, NY) mounted to an aluminum T-slot extrusion frame (80/20 Inc., Columbia City, IN) with two softbox photography lights (Neewer; Shenzen, China), four 70-watt CFL bulbs in total, to provide consistent illumination. Together with a connected laptop computer, the entire system was assembled on a utility cart (Uline, Pleasant Prairie, WI) creating a small, mobile imaging station. Images were captured via a laptop computer using Smart Shooter 3 remote capture software [53] allowing for automatic image naming via the affixed barcode, optimizing time and reducing human transcription error.

Root phenotyping software, ARIA 2.0 [52], was used to batch process over 12,000 images. Prior to image processing, color thresholding app extension in MATLAB (MathWorks, Inc., Natick, MA) was used to interactively develop a rule to generate a binary image for segmenting the foreground (root) and the background (blue germination paper). The developed rule was to convert the RGB image to HSV and consider the pixels with a hue (*H*) value higher than 175° as the blue background. The rule did not work for some images where the background was infrequently oversaturated by light reflection. In these cases, the RGB image was converted to Lab color space and a threshold value for L (lightness) channel was selected heuristically for the background. These rules were implemented in the ARIA 2.0. Bulk image sets were automatically processed through the ARIA 2.0 software for root segmentation and skeletonization, followed by root system architecture trait information extraction (Table 1). Seedling shoot and root dry weights were also collected at 12 days after germination.

### 2.2. Plant Materials

The diversity panel used in this experiment consisted of plant introductions (PIs) from the USDA core collection and Soybean Nested Association Mapping (SoyNAM) parental lines [54]. Selections from the SoyNAM panel included lines with diverse ancestry (*n* = 10) and high-yielding elite lines (*n* = 13), which were combined with the USDA core collection landraces (*n* = 269) to assemble a genetically diverse panel that has previously been genotyped [17] (Supplementary files 1 and 2). These genotypes consisted of a wide range of geographies (12 countries of origin), maturity groups (groups 1 (*n* = 19), 2 (*n* = 115), 3 (*n* = 156), and 4 (*n* = 2)), and growth habit (determinate (*n* = 88), semideterminate (*n* = 34), and indeterminate (*n* = 164)) along with various other morphological and seed quality traits.

### 2.3. Seedling Growth

The protocol for seed germination and transplanting is described by Falk et al. [52]. Ten seeds of each genotype were germinated in paper rolls in which two of the ten seedlings were transplanted at five days onto wet blue germination paper for further growth and root trait phenotyping. Two blue germination papers (30cm × 45cm) (Anchor Paper, Minneapolis, MN); each containing seedlings of each genotype was placed together, attached with binder clips as a growth pouch unit. Each growth pouch unit was suspended by the rungs of a growth chamber shelf with the lower 3 cm of the paper submerged in water as a wick to bring moisture to the roots.

The growth chambers were 175 cm by 100 cm and contained standard metal grate shelves 1.3 cm by 35 cm slots (Controlled Environments Ltd., Winnipeg, Canada). The growth chambers contained a plastic tote on the floor providing a water depth of 5 cm allowing each growth paper unit to be submerged to 3 cm. Growth chambers were set at 25°C during a 16-hour day, 22°C for an 8-hour night. Growth chamber light intensity was measured at 300 and 350 *μ*mol photons (m^−2^ s^−1^) using a Li-250A light meter (Li-Cor Biosciences, Lincoln, NE, USA).

### 2.4. Statistical Analysis

All statistical analyses were performed using R programming language unless otherwise specified. To evaluate the 292 genotypes, we first eliminated outliers using Tukey's boxplot method [55] before calculating the best linear unbiased predictor (BLUP) values for each trait utilizing a mixed model and the “lme4” package [56, 57]. In the model (Equation (1)), *y*_*ik*_ is the response variable of the *i*^th^ genotype at the *k*^th^ block (i.e., growth chamber used), *μ* is the total mean, *g*_*i*_ is the genetic effect of the *i*^th^ genotype, *b*_*k*_ is the block effect, and *e*_*ik*_ is the experimental error following *N*(0, *σ*_*e*_^2^). All factors were considered random effects. Broad sense heritability was calculated on an entry-mean basis using Equation (2), where *σ*_*g*_^2^ is the genotypic variance and *r* is the number of replications (*r* = 14). Tukey's honestly significant differences (HSD) [58] were used to identify statistical differences between genotype-based clusters (GBC) in which MSE is the mean squared error, *q* is the test statistic found in the *q*-table, and *S*_*a*_ is the number of observations of the *a*^th^ group (Equation (3)). To identify excessive correlation, a collinearity test of the predictor variables was performed using a variance inflation factor of five as a threshold to quantify the severity; *R* is the regression coefficient of the *j*^th^ variable with respect to the rest of the variables (Equation (4)). Fixation indices were calculated using the Hudson *F*_ST_ approach using the fst.hudson function in the KRIS R package where *n*_*i*_ is the sample size and ${\tilde{p}}_{i}$ is the sample allele frequency in population *i* for *iϵ*{1, 2} (Equation (5)).

(1) $$\begin{array}{c} y_{ik}=u+g_{i}+b_{k}+e_{ik}, \end{array}$$

(2) $$\begin{array}{c} H^{2}=\frac{\sigma_{g}^{2}}{\sigma_{g}^{2}+\left(\sigma_{e}^{2}/r\right)}, \end{array}$$

(3) $$\begin{array}{c} \text{Tuke}{\text{y}}^{\prime }\text{s HSD}=q\sqrt{\frac{1}{2}\text{MSE}\left(\frac{1}{S_{a}}+\frac{1}{S_{a`}}\right)}, \end{array}$$

(4) $$\begin{array}{c} \text{Variance inflation factor}=\frac{1}{1-R_{j}^{2}}, \end{array}$$

(5) $$\begin{array}{c} F_{\text{ST}}^{\text{Hudson}}=\frac{{\left({\tilde{p}}_{1}-{\tilde{p}}_{2}\right)}^{2}-\left(\left({\tilde{p}}_{1}\left(1-{\tilde{p}}_{1}\right)\right)/\left(n_{1}-1\right)\right)-\left(\left({\tilde{p}}_{2}\left(1-{\tilde{p}}_{2}\right)\right)/\left(n_{2}-1\right)\right)}{{\tilde{p}}_{1}\left(1-{\tilde{p}}_{2}\right)+{\tilde{p}}_{2}\left(1-{\tilde{p}}_{1}\right)}. \end{array}$$

### 2.5. Informative Root (iRoot) Categories

**Figure 1:**
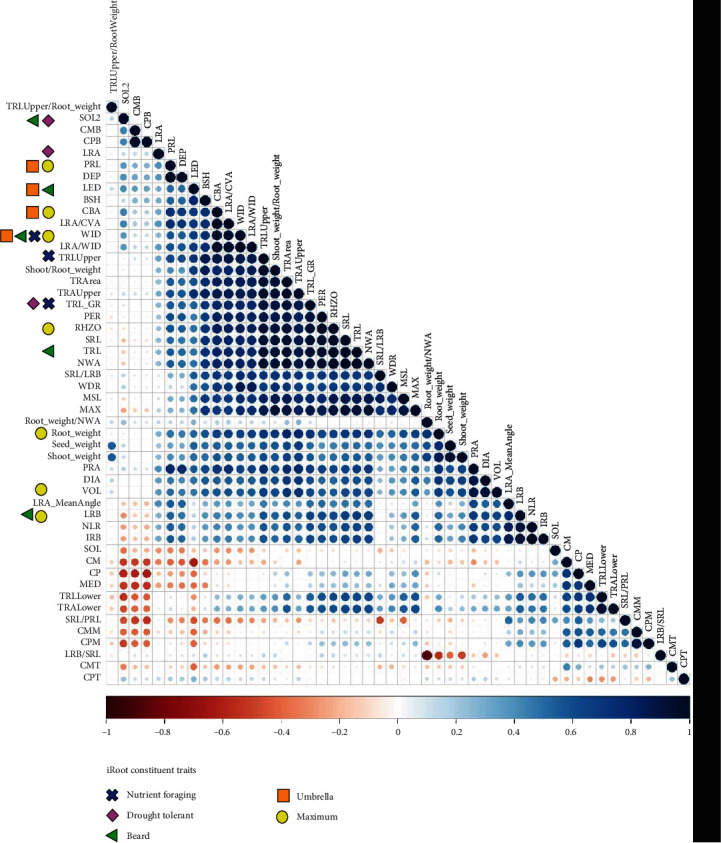
Pearson's correlations of root traits. Correlations at 9 days after germination measured on 292 soybean genotypes (replications = 14). Hierarchical clustering was used to group similar traits. Symbols (shape and color) denote RSA traits used in the corresponding iRoot index (cumulative trait scores).

We created iRoot categories in an effort to (a) reduce dimensionality (looking at a category rather than individual traits), (b) aggregate traits, as opposed to focusing on an individual root trait for increased biological relevance, and (c) identify specific trait measurements and statistical analyses to quantify differing root shapes based on a previous scientific work found in the literature [7, 9, 59–61]. All 292 genotypes were ranked from 1 (highest numerically) to 292 (lowest) based on each root trait. These rank scores of genotypes are summed for the specific traits that compile each iRoot category. Five iRoot categories and their constituent root traits included the following: (1) nutrient foraging (WID, TRL-GR, and TRLUpper), (2) drought tolerant (PRL, LRA, SOL2, and TRL_GR), (3) umbrella (PRL, WID, CVA, LRA, and LED), (4) beard (TRL, WID, LRB, LRA, SOL2, and LED), and (5) maximum (TRL, PRL, WID, CVA, LRB, VOL, RHZO, and Root_weight) (Figure 1). For example, a particular genotype ranked highly in the nutrient foraging iRoot category would display high values in three root traits: WID, TRL_GR, and TRLUpper. To be clear, iRoot categories selected roots that display root trait characteristics affiliated with the quality (e.g., deep roots for drought tolerance) in the growth chamber experiment, not to be confused with genotypes displaying “classical drought tolerance characteristics” in the field environment. Additionally, the analysis of all iRoot categories was restricted to images from nine days after germination (9 d) as particular slow growing genotypes lacked significant lateral roots at 6 d while other fast growing genotypes were subject to outgrow the germination paper medium by 12 d.

The nutrient foraging iRoot category was based from previous reports [36] which describe this phenotype as maximizing the distribution of lateral roots in the topsoil at a minimal metabolic cost to outperform competitor genotypes in nutrient poor soil. The phenotype was created to contain a wide root system with a high ratio for total root length in the upper 1/3 of the root system as well as a fast TRL_GR. The drought tolerant iRoot category followed the steep, deep, and cheap paradigm [43] created by selecting a long primary root with a high total root length growth rate while selecting steep lateral root angles (low angle is more advantageous) and minimizing root solidity (NWA/CVA) thus minimizing spatial density and therefore the metabolic cost. The beard iRoot type [62] maximizes total root length, the number of lateral root branches, and length distribution (TRLUpper/TRLLower) while minimizing total root width, lateral root angle, and root solidity (NWA/CVA) thus maximizing root density. The umbrella category [62] maximizes primary root length, root width, convex area, shallow lateral root angle, and length distribution (root length in the upper 1/3 over root length in the lower 2/3 of the root system). Finally, the maximum iRoot category was created for this study as an effort to identify genotypes that maximize the phenotypic potential without a particular environment in mind. As previously stated, these iRoot categories were created for dimension reduction, to increase of biological relevance and to facilitate comparison with a previous scientific work.

### 2.6. RSA Trait Correlations

Correlations between 49 plant traits were obtained using Pearson's correlation by implementing cor function in the “stats” package. Traits were grouped using hierarchical clustering using complete linkage with the hclust function. Visualization was performed using the “corrplot” package.

### 2.7. Phenotype-Based Clusters

Aside from typical trait-based analytical approaches, we explored alternate methods to group and correlate genotypes and their root trait phenotypes. While iRoot traits were picked using a heuristic approach, PBC were generated using unsupervised hierarchical clustering. Genotypes were grouped into eight PBC clusters based on 13 phenotypic root trait values (TRL, PRL, WID, CVA, LRB, VOL, LRA, SOL2, LED, RHZO, TRL_GR, TRLUpper, and Root_weight). A heatmap was created using the “heatmap2” and “dendextend” packages to display the interactions between the genotypic relationships and phenotypic trait values across the 13 root traits. In this manuscript, better performance indicates a higher value of the root trait, with LRA and SOL2 being exceptions. These are exceptions as steep root angle (lower number) and low solidity can be advantageous root traits.

### 2.8. Genotype-Based Clusters

Genotype-based clusters were formed by grouping the 292 genotypes into eight groups based on hierarchical clustering of the SNP data. These accessions, from the USDA soybean germplasm collection, have been genotyped using the Illumina SoySNP50k iSelect BeadChip (Illumina, San Diego, USA), which detected the segregation of 42,509 SNPs [51]. Using preprocessing steps to eliminate SNPs below a minor allele frequency of 0.05 and monomorphic SNPs, 35,448 SNPs were identified and used for subsequent analysis. Principal component analysis (PCA) was performed on SNP data using the prcomp function the “stats” package and graphed using the “ggplot2” package. Nei's genetic similarity was used to construct a pairwise distance matrix using all polymorphic SNPs [63–65]. Hierarchical cluster analysis using Ward's minimum variance produced a linkage dendrogram using the “dendextend” and “circlize” packages [66]. The optimal number of SNP-based clusters was determined using the iterative k-means approach in which the procedure successively increases the number of clusters and measures the goodness of fit based on the Bayes Information Criterion (BIC). Eight genotype-based clusters (GBC) were inferred from the inflection point in the BIC curve (Supplementary Figure S1).

### 2.9. Mean Root Shape and Shape Clusters

Shape-based clusters were formed by grouping the 292 genotypes into eight groups based on the mean root shape of each genotype, with clustering of the root shapes via a CNN algorithm. Mean root shape outline was generated from all the root images at day 9 for each genotype using Elliptical Fourier Transformation (EFT) [67] (Supplementary Figure S2). In brief the steps are as follows: (a) collect all the segmented root images for a genotype, (b) dilate the images with a 50 × 50 kernel, (c) extract the root shape outline, (d) perform EFT on the outline, (e) calculate mean Fourier descriptors for all the roots (*n* ≤ 14) for a genotype, and (f) reconstruct mean shape outline with N Fourier harmonics. Here, a dilation step (b) was necessary to capture the root shape faithfully by EFT. Additionally, in the reconstruction step (f), we used *N* = 5 harmonics to capture only the basic shape of the roots. Shape-based clustering was performed on the mean root shape using a CNN and k-means algorithms. In brief, the steps are as follows: (a) fill the mean shape outline and pad the image along the width direction to make it square, (b) use a convolution autoencoder network to convert the images into eight-dimensional (high-level feature) vectors (Supplementary Figure S3), and (c) cluster the roots into eight groups using k-means clustering with Euclidean distance as the distance metric.

## 3. Results

### 3.1. Genetic Diversity for Root System Architecture (RSA) Traits

Descriptive statistics, broad sense heritability and ANOVA analysis of root, shoot, and seed traits are reported (Table 2). Accessions displayed a range of phenotypic expression across the three imaging days. Representative phenotypic root traits for a particular maturity group, growth habit, or diversity was not identified. Genotypes were a significant source of variation for all but one trait (width-depth ratio (WDR) at 12 d). Large variation was observed for a majority of traits evidenced through comparison of mean, median, and trait ranges for RSA traits. Broad sense heritability (*H*^2^) across RSA traits ranged 0.26–0.93 (6 d), 0.14–0.92 (9 d), and 0.04–0.93 (12 d). Minimal differences of the traits were observed among the diverse, elite, and landrace groups. Supplementary Table S1 displays the statistically significant root traits between diversity groupings. PRL, PRA, and DEP of accessions with diverse ancestry, and elite lines were often larger than landraces at 9 d and 12 d based on Tukey's HSD metrics. Additionally, WDR of landraces at 12 d was higher than elite lines which could be attributable to elite lines' increased DEP. No perceptible relationship patterns were detected among genotypes when comparisons were made between maturity groups and growth habits. These relationships were explored using tSNE and Gower clustering approaches, as well as neural networks.

### 3.2. Trait Relationship and iRoot Categories

Trait relationships were determined using Pearson's correlation coefficients (Figure 1). A large set of traits, which provide general metrics for length, width, and area (WDR, MSL, SRL_LRB, Area, TRLUpper, TRAUpper, MAX, TRL_GR, PER, RHZO, SRL, TRL, and NWA), showed strong correlations and formed a single hierarchical cluster. Root_weight correlated (greater than 0.8) with shoot weight, TRArea, and TRAUpper. TRArea and TRAUpper formed a separate cluster together. Intertrait correlations were strongest at 6 d and decreased in intensity (measured using cumulative correlation intensity) in successive stages (Supplementary Figure S4). Individual traits often transitioned among clusters between time points; however, the general clustering of traits related to (a) length, (b) width, and (c) area remained consistent.

**Figure 2:**
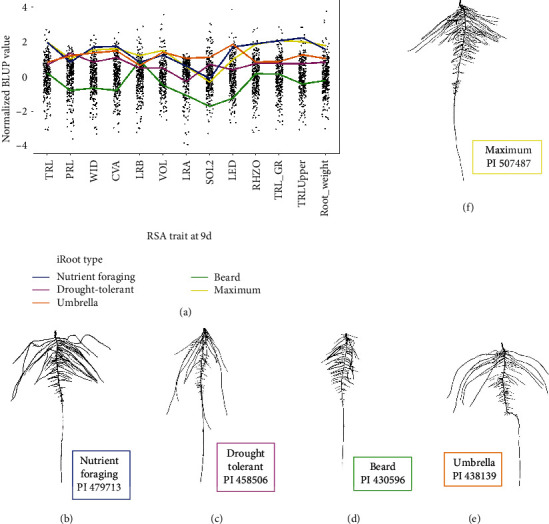
Five iRoot categories developed using 13 descriptive traits. (a) Root trait performance generated from the mean trait values of the top 10 ranked genotypes based on iRoot metrics at 9 days after germination. Data was compiled from 292 soybean accessions (replications = 14; genotypic BLUPs are represented by the black dots). Segmented root images of the top ranked genotypes representing the five iRoot categories: (b) nutrient foraging, PI 479713; (c) drought tolerant, PI 458506; (c) beard, PI 430596; (d) umbrella, PI 438139; and (e) maximum, PI 507487 displayed at 9 days after germination.

Due to the close correlation of many of the root traits, from here on in this paper, we narrow the focus to 13 root traits (TRL, PRL, WID, CVA, LRB, VOL, LRA, SOL2, LED, RHZO, TRL_GR, TRLUpper, and Root_weight) that define the iRoot categories. While some of the 13 root traits showed collinearity, we report on all to ensure comparison with previous literature [7, 9, 59–61] (Supplementary Table S2). These thirteen root traits show a range of phenotypic expression (Figure 2). For effective visualization and analysis, mean values of the traits for the top 10 ranked iRoot genotypes were employed as a reference (Figure 2, Table 3).

The highest ranked soybean genotypes in each of the iRoot category were as follows: nutrient foraging—PI 479713, drought tolerant—PI 458506, umbrella type—PI 438139, beard type—PI 430596, and maximum—PI 507487 (Figure 2). Particular accessions often scored high rankings in two or three different iRoot categories. For example, genotypes PI 507487 and PI 578367 ranked in the top ten soybean lines for three separate iRoot categories including maximum, nutrient foraging, and umbrella. Soybean genotypes PI 458506 and PI 507491 scored in top ten for maximum, nutrient foraging, and drought tolerant, while PI 89134 placed in top ten for maximum, beard, and drought tolerant categories (Supplementary Figure S5). The maximum and foraging iRoot categories have a substantial overlap (7 of the top 10) of genotypes representing both categories. These results reveal that despite being calculated from different root traits, iRoot categories such as maximum, nutrient foraging, and umbrella displayed similarities due to trait correlations.

### 3.3. Phenotype-Based Clusters

Aside from typical trait-based analytical approaches, we explored alternate methods to group and correlate genotypes, their genotypic data, root shape, and individual root trait phenotypes. Eight phenotype-based clusters (PBC) were created based on hierarchical clustering of thirteen root traits used to create the iRoot categories. Due to the nature of the analysis and its reporting, PBC were arranged so that high performing genotypes constituted PBC “A” (*n* = 3) while a decreasing gradient was formed to the lowest performing PBC “H” (*n* = 4) (Table 4, see also Supplementary Table S3). The majority of the 292 genotypes fell into PBC “C,” “D,” and “E” creating a bell-like distribution curve from PBC 1 to PBC 8. One exception to the decreasing gradient was the SOL2 root trait and, to a lesser extent, LRA. Lower angles provide a steeper root angle of attack, and such genotypes can cover a large area with limited root length scavenging soil more efficiently.

### 3.4. Genotype-Based Clusters

**Figure 3:**
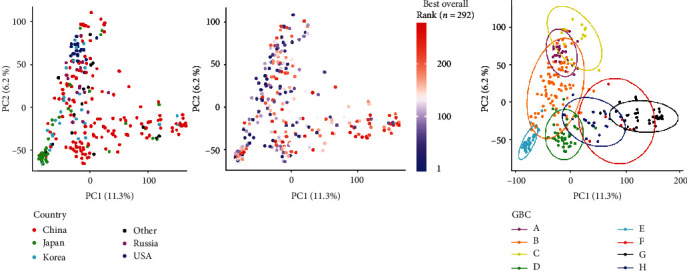
Principal component analysis of 292 soybean genotypes. Genotypic data 35,448 SNP markers. The two principal components accounting for 11.3% (PCA1) and 6.2% (PCA2) of the genetic variation. (a) Color represents the country of origin. (b) Color represents “maximum” iRoot category rank. Blue (best rank) to red (worst rank) color gradient is used to show ranks of 292 soybean genotypes. (c) 292 genotypes colored by their allotted genotype-based cluster (GBC).

Genotype-based clusters were formed by grouping the 292 genotypes into eight groups based on clustering the SNP data. The first two principal components explain 11.3% and 6.2% of the genetic variation (Figure 3). Visually, the PCA scaffold was predominately soybean accessions from China, while clusters of accessions representing USA, Japan, and Russia were also evident (Table 4). To further visualize the relationship between country of origin and GBC, we created a dendrogram representation based on genetic distance and the genetic clustering of the 292 soybean genotypes (Supplementary Figure S6). The USA accessions comprised a majority of GBC “A,” Japan in “E,” and Korea and Russia in “B.” Soybean accessions from China were represented in all eight GBC. GBC “B” and “E” accessions were primarily of determinate growth habit. Mean fixation indices, based on SNP values, were calculated between GBC to display genetic diversity (Table 5). The ranking of iRoot categories showed that GBC “B,” “C,” “D,” “E,” and “H” have representatives in the top 25 representative genotypes of each iRoot categories while GBC “F” has none (Supplementary Table S5).

### 3.5. GBC-PBC Relationship

**Figure 4:**
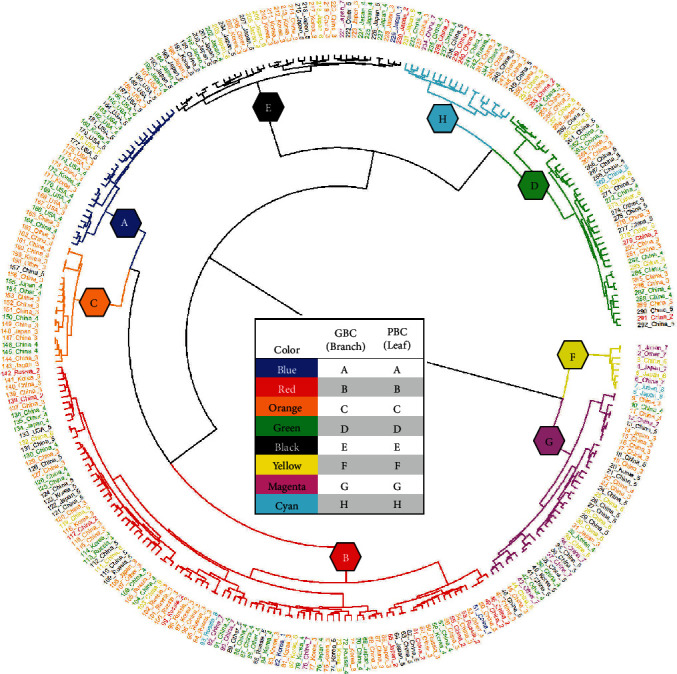
Dendrogram of genomic and phenomic relationships of 292 soybean genotypes. Eight genotype-based clusters (GBC) based on genetic distances are represented in the tree's branch position and colors. Eight correlating phenotype-based clusters (PBC), based on complete linkage of 13 root traits (TRL, PRL, WID, CVA, LRB, VOL, LRA, SOL2, LED, RHZO, TRL_GR, TRLUpper, and Root_weight) at 9 days after germination, are represented as the tree's leaf colors. Genotype country of origin is displayed in the tree's leaf text.

To visualize the relationship between PBC and GBC, we created a dendrogram representation containing PBC and GBC information of the 292 soybean genotypes (Figure 4). The distance between the tree's branches is based on genetic relatedness, the branch color represents the GBC of the genotypes, the leaf text denotes the country of origin, and the leaf text color represents the PBC of the genotype. A phenotype-based clustering algorithm created a gradient from high to low root trait values. The first PBC with genotypes having numerically high trait values we labeled “A,” while the last PBC with very low trait values we labeled “H.” PBC “A” was given a numerical value as “1” with a gradient of reducing root trait values to PBC “H” as “8.” These numerical values allowed us to correlate GBC and PBC. We ranked the grouping of each GBC by calculating the mean PBC of the genotypes within the GBC cluster: 1 being high and 8 being low. Genotype-based clusters with a PBC mean closer to 1 correspond to genotypes demonstrating high root trait values, while a mean closer to 8 suggests low trait values. The resulting mean PBC values for each GBC from “A” to “H” are 4.00, 3.72, 3.35, 4.15, 4.12, 6.88, 4.55, and 3.7, respectively (Table 4). Genotype-based clusters with a mean < 4: “B” (3.72), “C” (3.35), and “H” (3.7), are comprised of genotypes with numerically higher trait values. Genotypes comprising GBC “F” and “G” display mean PBC values of 6.88 and 4.55 consisting of lower root trait values.

To correlate between PBC and country of origin, the mean PBC value for each country, from low to high PBC mean, was 3.47 for Russia, 3.65 for Korea, 3.99 for China, 4.17 for USA, 4.29 for Other, and 4.46 for Japan. To correlate between iRoot categories and PBC, we focused on PBC values of only the top 10 genotypes of each iRoot category. The top 10 genotypes of the maximum and drought tolerant categories come from high performing PBC “A,” “B,” and “C” while the top 10 nutrient foraging came solely from PBC “A” and “B.” The top 10 genotypes of the umbrella type representatives are from mid-performing PBC “B,” “C,” and “D”; and the beard type has 10 representatives in relatively lower performing PBC “C,” “D,” and “E.”

**Figure 5:**
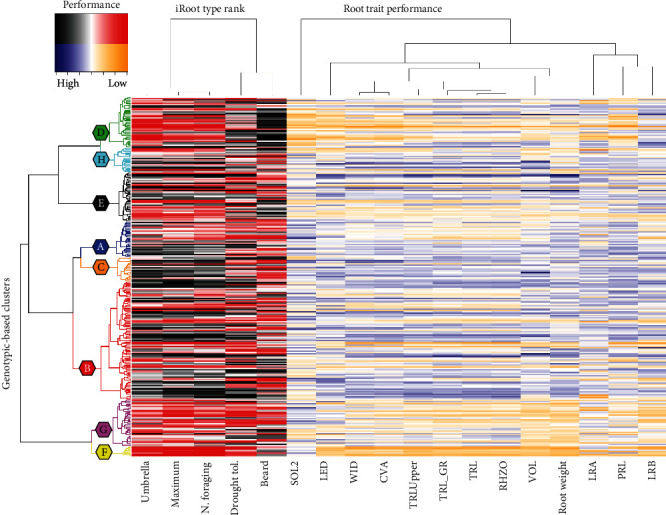
Heatmap displaying relationships between genotypes and phenotypes. Correlations between genotype (*y*-axis) and phenotype (*x*-axis) for 9 d after germination using data from 292 soybean accession studies for 13 root traits (TRL, PRL, WID, CVA, LRB, VOL, LRA, SOL2, LED, RHZO, TRL_GR, TRLUpper, and Root_weight) and 5 iRoot categories (nutrient foraging, drought tolerant, umbrella, beard, and maximum) (replications per accession = 14). The dendrogram on the *y*-axis was developed using hierarchical clustering using SNP data; the *x*-axis displays iRoot categories and 13 root traits that comprise iRoot categories.

A two-way clustering heatmap was used to further illustrate the comparison of genotype and phenotype performance (Figure 5). The dendrogram tree on the left-hand side groups the 292 genotypes based on genetic relatedness with GBC identified by color. The first branch division of the tree indicates that GBC “G” and “H” are genetically distinct from the other 6 GBC. Genotypes from these to clusters have also contained lower root trait values (Table 4). On the red-black heatmap to the right of the dendrogram, color is based on iRoot category with black signifying high (closer to 1) and red signifying low (closer to 292) ranking for the iRoot category. Genetically distinct GBC “G” and “H” show poor ranking (red) across most of the iRoot categories with exception of the beard type. GBC “C” displays high root trait values (black) for three iRoot categories. Other GBC such as “A,” “B,” and “E” are definitive with branches within each cluster containing high values with others low. A second dendrogram above the heatmap connects phenotypic traits based on hierarchical relatedness of their numerical values. Here, the iRoot categories of umbrella, maximum, and nutrient foraging form a distinct group. The largest grouping of root traits consisted of WID, CVA, TRLUpper, TRL_GR, TRL, and RHZO; and this group remained consistent at 6 d, 9 d, and 12 d while the remaining 7 traits did not form tight groups (Supplementary Figure S7).

The heatmap on the right-side is based on phenotypic performance of 13 root traits with blue being high and orange being low trait values. GBC “F” and “G” were generally grouped together as identified by the red (left) and orange (right) shading denoting low value iRoot category performance. Conversely, GBC “B,” “C,” and “H” perform well with black (left) and blue (right) shading. Further examination within each GBC indicates that particular subbranches within each cluster often show higher values than others which is evident for iRoot categories and individual traits. For example, this is evident in the lowest-most subbranch of the large GBC “B.” This subbranch contains 19 genotypes which display high values for umbrella, maximum, nutrient foraging, and drought-tolerant iRoot types as well as for many root traits.

To further explore the relationship between genotypic data, phenotypic data, and iRoot categories, iRoot category ranking (for each genotype from 1 to 292) was averaged for each GBC (Table 4). The results reflect whether certain genotypic clusters display iRoot features. In step with earlier results, GBC “C” produced the highest average ranking in umbrella, nutrient foraging, and maximum iRoot types as well as having the highest overall average ranking (116 of 292) (Table 4). GBC “F” produced the worst ranking in nutrient foraging, drought tolerant, umbrella, and maximum iRoot types as well as the worst overall average ranking (239 of 292). Ranking results for GBC “D” displayed a high ranking for the beard iRoot type (68 of 292). Interestingly, the average iRoot scores of the 19 genotypes that make up a small, aforementioned genetically divergent subbranch of GBC “B” are 89, 104, 66, 95, and 164 out of a possible 292 for maximum, drought tolerant, nutrient foraging, umbrella, and beard iRoot types, respectively. These results suggest that these 19 genetically related accessions display many desirable phenotypic attributes in this experimental environment. Genotypes from Russia and Korea produced higher than average iRoot rankings (121 of 292) each in relation to China = 147, Other = 148, USA = 159, and Japan = 164. Finally, maximum root type iRoot rank results were plotted on the genomic data that produced PCA plot for visualization (Figure 3(b)). The presence of plane separation between red (low root trait values) and blue (high root trait values) data points displays the evidence of the correspondence between the performance of soybean accessions and genotypic data.

**Figure 6:**
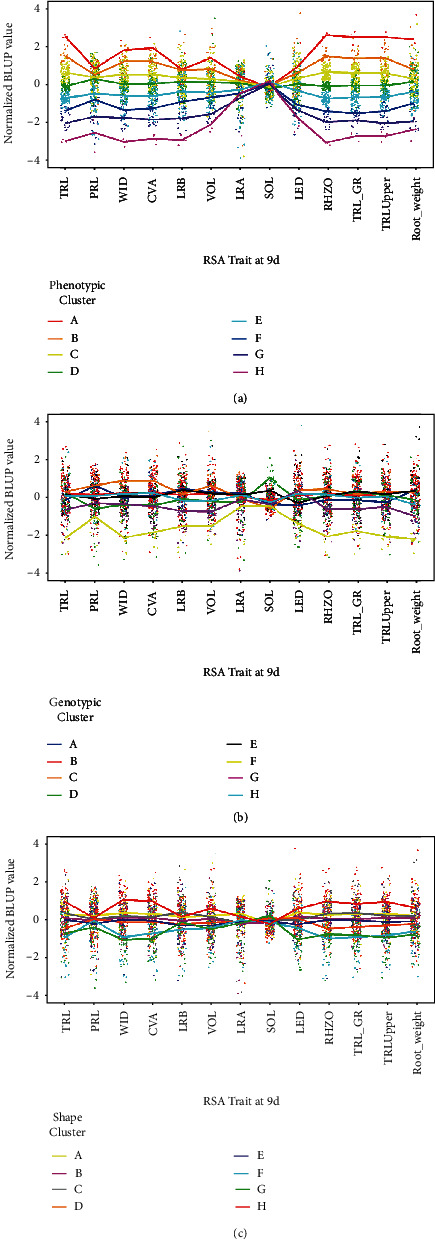
Line graphs of phenotype-, genotype-, and shape-based clusters. Scaled mean value of 13 RSA traits (TRL, PRL, WID, CVA, LRB, VOL, LRA, SOL2, LED, RHZO, TRL_GR, TRLUpper, and Root_weight) at 9 days after germination of (a) eight genotype-based clusters (GBC) based on genetic distances and hierarchical cluster analysis, (b) eight phenotypic-based clusters (PBC) based on complete linkage of 13 root traits and, and (c) shape-based clusters (SBC) based on Euclidean distances among the root shape outlines represented in an eight-dimensional high level feature space. Data was compiled from 292 soybean accessions; 14 replications; genotypic BLUP values are represented by colored points.

Differences appear when comparing genotype- and phenotype-based clustering methods across 13 root traits in which GBC “C” performed well and GBC “F” performed poorly (Figures 6(a) and 6(b)). Genotypic-based clusters “B,” “E,” and “H” display consistency in relation to the other clusters. The trend lines from 6 d to 12 d show that GBC “F” and “G” consistently have lower trait value performance (Supplementary Figure S8). GBC “A” performed similarly to GBC “B,” “C,” and “E” for the majority of root traits with the exception of PRL and DEP. PRL and DEP have a higher trait value compared to the mean at 6 d, 9 d, and 12 d. The LRB trait has 1.1% more lateral root branches than the mean on 12 d (Supplementary Table S4). In GBC “A,” with the exception of LG05-4464, the 23 genotypes from the USA displayed relatively similar trait values to one another (data not shown). LG05-4464, a line with diverse ancestry, ranked first of 23, in TRL and TRArea measurements as well as WID, and thus outpaced the others within the USA-dominated GBC “A.”

### 3.6. Shape-Based Clusters

Shape-based clustering into eight groups was performed on 9 d roots. Mean values of shape-based clusters (SBC) labeled “A,” “B,” “C,” and “D” display high values across the 13 root traits while SBC “G” and “H” display low values (Figure 6(c)). SBC “A” has high values in the 13 traits used in iRoot estimation while SBC “H” has generally low values (Table 4). Cross-referencing SBC “A” to GBC, individuals were derived from higher trait valued GBC including GBC “B” (*n* = 17), “H” (*n* = 6), “C” (*n* = 4), “D” (*n* = 4), and “E” (*n* = 4). GBC “F” contains eight genotypes which all correlate to low trait valued shape clusters, four being in SBC “G” and four being in SBC “H”. SBC “A” has a higher iRoot ranking than other SBCs for maximum, drought tolerant, nutrient foraging, and umbrella types while SBC “H” has the highest ranking for beard type (Table 4). Accessions from China are well distributed across SBC while accessions from Japan have a moderate presence in SBC “H” (*n* = 14). Genotypes derived from the USA-based cluster (GBC “A”) do not show distinct correlation with SBC, however, lacking presence in both high (SBC “A”) and low (SBC “H”) root trait valued clusters.

### 3.7. Mean Root Shape Outline of the iRoots

**Figure 7:**
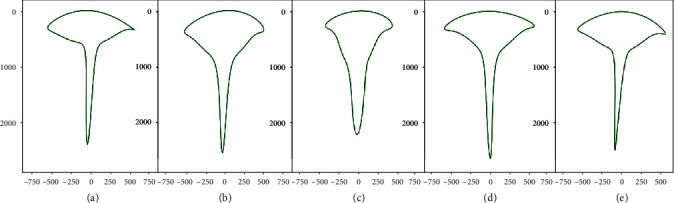
Root profiles of five iRoot categories. Images at 9 d were collected of the top 10 ranked genotypes of each iRoot category using the mean boundary with Fourier coefficients at five harmonics: (a) nutrient foraging type (*n* = 92), (b) drought tolerant (*n* = 86), (c) beard type (*n* = 98), (d) umbrella type (*n* = 100), and (e) maximum (*n* = 89). Axis units are in pixels. To facilitate comparisons, all the shapes were aligned at the top (at *r* = 0) and were vertically centered (*c* = 0).

Mean root shape profiles of the top 10 ranked genotypes of each iRoot at 9d were generated using five harmonics of Elliptic Fourier descriptors as described in Materials and Methods (Figure 7). The shape profiles were created solely from images of the top 10 ranked genotypes of each iRoot category. Initial impression suggests the outlines look similar; however, each profile is subtly distinct due to a combination of shape, size, and structure. The nutrient foraging and maximum representations display a similar shape while the beard shape is visually distinct from the other categories. The drought-tolerant based shape shows a reduced upper to lower width ratio compared to maximum, umbrella, and nutrient foraging which were less distinct from each other.

## 4. Discussion

Over 12,000 images were collected over three time points throughout the course of this experiment. ARIA 2.0-based trait extraction provided over 500,000 data points. The best linear unbiased predictors (BLUPs) were calculated to act as a weighted genotypic mean for all root system architecture traits as well as seed, root, and shoot dry weights. Broad sense heritability of root traits was equal or above similar previous studies [7, 59, 61] with 15 traits being above 0.9 and 27 being above 0.8. These heritability results could be reflective of the number of replications (14) used in this study as compared to previous studies (Pace et al., *n* = 3; Adu et al., *n* = 5; Gioia et al., *n* = 10; Liu et al., *n* = 8; 15) [7, 59, 61, 68]. The inherent variability in complex root traits requires maximizing replications. Our analysis indicated that 14 replicates maximized broad sense heritability and are advantageous to lower the number of replications (Supplementary Figure S9). Strong correlations between root traits concurred with an a priori hypothesis that Root_weight correlated with long TRL and increased LRB, CVA, VOL, and WDR. The root traits providing metrics for length and width generally clustered together, while those for weight, volume, and root number congregated together. Additionally, correlations between traits exhibited the highest values (measured using cumulative correlation intensity) at 6 d and lowest at 12 d. This effect could be the result of lower trait variability at earlier growth stages.

This study is aimed at connecting genomic and phenomic information to elucidate genetic diversity present for RSA traits. Using genomic distance approaches, genotypes were grouped into eight clusters for analysis using SNP data (GBC) and eight clusters using the phenotypic data (PBC) using data from 13 root traits. We explored the trait expression in depth to determine the extent of genetic diversity as it is important for breeding applications. Selection efforts in the last century have only indirectly targeted root architecture traits, while the primary efforts have been to select for seed yield under the influence of agronomic and management practices including plant population density, fertilizer application, water availability/irrigation, and soil types. Implicit assumptions are made that above ground trait variability and expression are mirrored in below ground RSA traits. This knowledge could be leveraged in the future as studies have shown a positive relationship between a common bean leaf surface area and a root surface area [69], allowing for indirect selection of root through shoot assessment. Our results show that GBC “A”, composed primarily of US accessions, did not have full expression of phenotypic diversity for RSA traits. GBC “A” at 9 d displayed average root trait expression with the exception of above average PRL and DEP (two correlated traits; at 9 d; *r* = 0.996) suggesting US cultivars exhibit increased depth suggesting potential drought tolerance characteristics. However, GBC “A” did not display differences in LRA or TRL_GR, with other traits reported for positive drought response. A field study is needed to ascertain if the US germplasm accessions have been indirectly selected for long PRL (reported in this paper using controlled environment conditions), which has been linked to increased drought tolerance [33, 34], and for LRA and other surrogates of drought tolerance response. Additionally, high root trait values such as TRL have not been correlated to increased seed yield in soybean. While some US accessions were cultivars, the lack of RSA trait diversity presents opportunities to further improve the genetic potential of soybean cultivars. While our interest is predominantly focused on studying the US accessions, these approaches and results can be useful for breeders and researchers worldwide to understand the full complement of genetic and trait diversity in their programs and targeted regions. Strong performing accessions can be identified individually or as a cluster using genomic population structure to direct further exploration using genomic selection with a multivariate approach bridging genomics and phenomics data. For example, subbranches within genomic-based clusters contained many highly related genotypes displaying high root trait values. The near-exhaustive analyses we performed did not uncover significant correlations to geographic origin, climatic zone, and root phenotype. Our scope of inference is limited to 6, 9, and 12 days after germination, and additional studies are needed to confirm if this lack of relationship is maintained at later growth stages.

**Figure 8:**
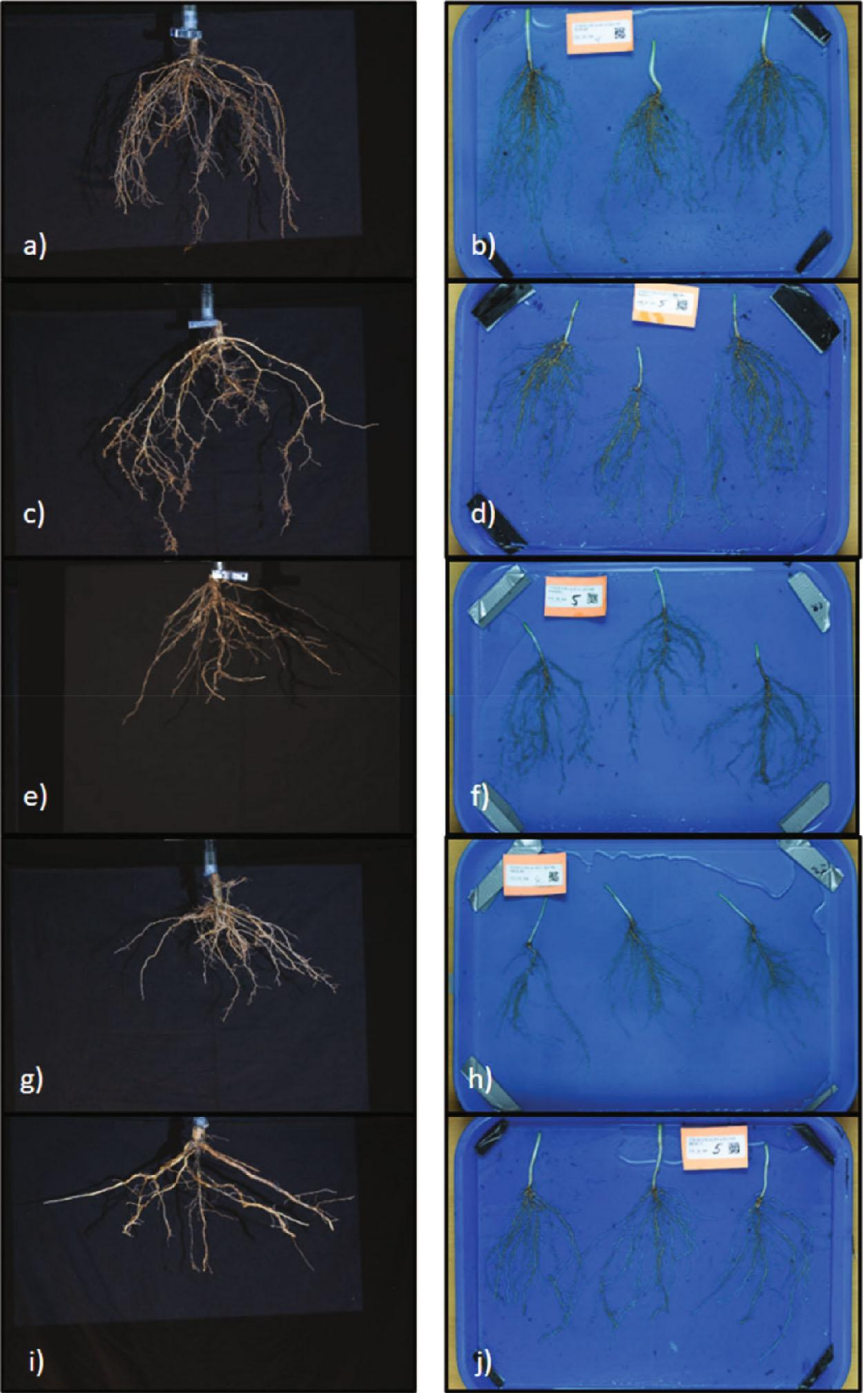
Field extracted roots. Images of iRoot representative roots excised from soil at the R7 (left) and V1 (right) stages. (a, b) maximum (PI 507487), (c, d) nutrient foraging (PI 479713), (e, f) drought tolerant (PI 458506), (g, h) beard type (PI 430596), and (i, j) umbrella type (PI 438139).

The iRoot categories were generated by leveraging phenomics data to identify specific trait measurements and statistical analyses to quantify differing root shapes. While these iRoot categories have been reported in different crops, we were interested in integrating information from multiple crops to study and explain the root trait diversity in soybean. The iRoot categories were based on a previous work of the root scientific community including nutrient foraging [36], drought tolerant [43], beard [62], and umbrella [62]. The maximum iRoot category was developed to identify the greatest root growth potential without a particular environment in mind. The umbrella iRoot category was based on Liao et al.'s study, who use the common bean as an archetype of umbrella shape describing it as P-foraging noting that “basal roots tend to be shallow in the phosphorus-rich topsoil and tap roots tend to be deep for water in the subsoil.” The nutrient foraging iRoot category, similar to the umbrella type, was created to capture a root phenotype that could optimize nutrient acquisition in low-fertility soils [36]. The nutrient foraging iRoot is composed of a wide root system with a high ratio for total root length in the upper 1/3 of the root system as well as a fast growth rate. The beard shape iRoot was noted as the ideal rice root type, “moderately dispersed yet uniformly distributed adventitious and lateral roots so as to keep most roots in the topsoil for phosphorus and a few roots in the subsoil for water” [59]. The drought tolerant iRoot category was developed to “chase the water”; in other words, fast growing, steep, deep roots provide yield security during drought [36, 49]. Potentially, soybean genotypes with a dominant, rapidly elongating taproot could lead to a deeper root system and enhanced water acquisition. Uga et al. report that steeper root angles in rice have also been correlated to higher yield in drought environments [70]. Our observation of the correlation between shallow LRA and TRL growth rate (*r* = 0.28) suggests that, genetically, roots may have a predisposition to both traits and require further testing in field tests. Lab-based root angles have been shown to correlate to drought tolerance by other studies [37, 71] including Rellán-Álvarez et al. who noted that water-deficient Arabidopsis roots grow at a steeper angle in soil-filled rhizotrons compared to the well-watered treatment and serve as an optimal starting point for larger scale genetic studies [9]. The maximum iRoot, which successfully correlated phenotypic root traits with genotypic based population structure visualized in Figure 3(b), suggests that genotypic information can predict certain population groups which may have potential use in breeding. The next step in this root trait research is to develop and perform a large-scale study to correlate controlled environment and field-based results. Our preliminary investigations of top ranked iRoot genotypes in a field environment experiment display visual similarities worth further investigation (Figure 8). Additional experiments are needed to verify the seed yield performance associated with different iRoot categories.

We combined mathematical functions Elliptical Fourier Transformation (EFT) and machine learning (ML) approaches on image data to generate shape profiles and shaped-based clusters, which have previously not been reported for root-related trait studies. The EFT approach is advantageous to explore root shape diversity allowing for systematic root outline analysis while maintaining the integrity of the shape. In our efforts, we generated mean shape profiles of the iRoot categories and the profiles qualitatively capture the expected difference among the categories. The shape-based clusters removed the human annotation steps and helped to segregate strong performing and weak performing genotypes into different shape-based clusters. SBC “A” contained genotypes with high mean trait values that were grouped into high performing PBCs. Additionally, the SBC “A” was also the highest ranking for 4 of 5 iRoot categories. Genotypes grouped within SBC “G” and “H” performed poorly for trait values, PBC, and iRoot categories. These results suggest that high performing genotypes can be identified solely from their root shape and present an attractive application for phenomics in breeding and research. However, this approach requires further validation for comparison with state-of-the-art shape profile generation and applicability to field performance through plant breeding efforts.

We propose that RSA trait research for practical breeding outcomes will benefit from further studies in high throughput phenotyping systems that can do the following: (a) connect artificial and field environment studies, (b) make correlations between easily assessed and difficultly assessed traits to determine optimal balance of traits to focus on, (c) understand the physiology behind drivers for yield using large plant populations in specific and diverse environments, and (d) integrate genomics and phenomics pipeline for breeding decision-making. Root trait research requires infusion of analytics, such as leveraging advanced sensors coupled with computer vision and ML-based feature extraction methods [72–77]. Finally, robotics-assisted phenotyping is transforming above ground trait studies [78], and there is a need for robotics-based phenotyping solution for end-to-end phenotyping platforms and root excavation without information loss. Unlike above ground traits, root systems still do not have well-described growth stages and are often described by corresponding length or depth which lacks the inclusion of development stages [79, 80]. An understanding of root growth stages and developmental process stages and processes is integral to adapting root development into mathematical growth models, which will help breeders develop more efficient crops. These abovementioned solutions will help integrate multitrait objective functions [81] for above and below ground trait selections for furthering genetic gains. There is a need to deploy RSA traits in prescriptive cultivar development [82] and continue to explore and identify trait predictors for phenomics-assisted breeding [83].

## 5. Summary

In this study, we explored informative root categories (iRoot), built on a previous literature in different crops, leveraging data to identify specific trait measurements and statistical analyses to quantify iconic root shapes. Results demonstrate that superior root trait values and root shape correlate to specific genomic clusters. In addition, US-derived genotypes have long primary roots but fail to show further root trait values indicating room for improvement of the RSA of US germplasm. However, other groupings of genotypically related accessions did show high root trait values. Our study demonstrates the relevance of ML and computer vision-based software for the study of RSA traits. These tools can be useful for discovering and characterizing new traits and advancing time series-based studies on the growth and development of root systems. While we now can correlate root values and shape to genomic clusters, there is a need to connect controlled environment studies to field-based studies to improve methods of data collection. There is a large inventory of genetic variation among the world's soybean germplasm collection which provides the base for future crop improvement for RSA traits. After centuries of indirect selection for RSA, there is a pressing need to harness and implement quality soybean RSA diversity in cultivar development programs. Building upon the correlations of root phenotype and shape to genomic regions with improved phenotyping and ML techniques, we have captured the diversity of RSA available within the soybean germplasm core collection and can now leverage our results to develop improved soybeans using traditional and phenomics integrated plant breeding approaches.

## Figures and Tables

**Table 1:** Measured and derived root system architecture traits captured by ARIA 2.0 including the 13 which were used to create iRoot categories and for clustering analysis.

Symbol	Trait name	Unit	Trait description
TRL	Total root length^∗^	Cm	Cumulative length of all the roots in centimeters
TRL_GR	Total root length growth rate^∗^	Cm	Change in total root length
PRL	Primary root length^∗^	Cm	Length of the primary root in centimeters
TRLUpper	Total root length upper^∗^	Cm	Total root length of the upper one-third
DEP	Depth^∗^	Cm	The maximum vertical distance reached by the root system
WID	Width^∗^	Cm	The maximum horizontal width of the whole RSA
CVA	Convex area^∗^	cm^2^	The area of the convex hull that encloses the entire root image
RHZO	Rhizosphere area^∗^	cm^2^	Length of 2 mm surrounding the TRL
VOL	Volume^∗^	cm^3^	Volume of the primary root
LRA	Lateral root angle^∗^	Angle	Median root angle along the extent of all lateral roots
SOL2	Solidity (inverse)^∗^	Ratio	The fraction equal to the convex area divided by the network area
LED	Length distribution^∗^	Ratio	TRLUpper/TRLLower
NLR	Nodes of lateral roots	Count	Number of nodes of lateral roots
IRB	Independent lateral root branches	Count	Number of independent lateral root branches
MED	Median	Count	The median number of roots at all Y-location
MAX	MaximumR	Count	The maximum number of roots at all Y-location
TRArea	Total root area	cm^2^	Area of the RSA as observed in the 2D projected view
PRA	Primary root surface area	cm^2^	Surface area of the primary root
TRAUpper	Total root area upper	cm^2^	Total root area of the upper one-third
TRALower	Total root area lower	cm^2^	Total root area of the lower two-thirds
WDR	Width/depth ratio	Ratio	The ratio of the maximum width to depth
SOL	Solidity	Ratio	The fraction equal to the network area divided by the convex area
BSH	Bushiness	Ratio	The ratio of the maximum to the median number of roots
SRL/PRL	SRL by PRL	Ratio	Number of the secondary root per unit length of the primary root
COM	Center of mass	Ratio	Center of gravity of the root/depth
COP	Center of point	Ratio	Absolute center of the root regardless of root length/depth
CMT	Center of mass (top)	Ratio	Center of gravity of the top 1/3 of the root (top)/depth
CMM	Center of mass (mid)	Ratio	Center of gravity of the middle 1/3 root (middle)/depth
CMB	Center of mass (bottom)	Ratio	Center of gravity of the bottom 1/3 root (bottom)/depth
CPT	Center of point (top)	Ratio	Absolute center of the root regardless of root length (top)/depth
CPM	Center of point (mid)	Ratio	Absolute center of the root regardless of root length (middle)/depth
CPB	Center of point (bottom)	Ratio	Absolute center of the root regardless of root length (bottom)/depth
	Seed weight	Grams	Weight of 100 seeds
	Shoot weight	Grams	Dry weight of shoot
	Root_weight^∗^	Grams	Dry weight of root

Pixels converted to cm. ^∗^Root trait used in one or more iRoot categories.

**Table 2:** Descriptive statistics. Broad sense heritability and ANOVA analysis of 38 RSA traits; plant and seed weights at 6, 9, and 12 days after germination. Data from 14 replicates were included in the analysis.

	Day 6								Day 9								Day 12							
	Mean	Median	Min	Max	SD	*H* ^2^	Genotype	SB	Mean	Median	Min	Max	SD	*H* ^2^	Genotype	SB	Mean	Median	Min	Max	SD	*H* ^2^	Genotype	SB
Seed weight	14.9	14.8	4.0	39.2	4.8				14.9	14.8	4.0	39.2	4.8				14.9	14.8	4.0	39.2	4.8			
Shoot weight	0.23	0.23	0.03	0.67	0.07	1.00	^∗∗∗^	^∗∗∗^	0.23	0.23	0.03	0.67	0.07	1.00	^∗∗∗^	^∗∗∗^	0.23	0.23	0.03	0.67	0.07	1.00	^∗∗∗^	^∗∗∗^
Root_weight	0.09	0.09	0.01	0.26	0.03	1.00	^∗∗∗^	^∗∗∗^	0.09	0.09	0.01	0.26	0.03	1.00	^∗∗∗^	^∗∗∗^	0.09	0.09	0.01	0.26	0.03	1.00	^∗∗∗^	^∗∗∗^
TRL	68.9	65.3	0.2	227.2	39.1	0.92	^∗∗∗^	^∗∗∗^	215.1	208.6	2.8	566.5	90.7	0.92	^∗∗∗^	^∗∗∗^	338.3	331.4	0.6	923.3	119.0	0.93	^∗∗∗^	^∗∗∗^
PRL	20.8	21.1	0.2	38.2	4.9	0.92	^∗∗∗^	^∗∗∗^	35.0	35.5	1.7	54.4	7.3	0.86	^∗∗∗^	^∗∗∗^	46.6	47.9	1.2	68.3	8.7	0.89	^∗∗∗^	^∗∗∗^
SRL	48.2	43.4	0.0	195.2	35.3	0.92	^∗∗∗^	^∗∗∗^	180.0	171.8	0.1	527.5	87.0	0.92	^∗∗∗^	^∗∗∗^	292.2	284.4	0.1	871.0	115.7	0.92	^∗∗∗^	^∗∗∗^
MSL	1.2	1.1	0.0	5.2	0.7	0.91	^∗∗∗^	^∗∗∗^	3.1	2.8	0.0	13.8	1.5	0.89	^∗∗∗^	^∗∗∗^	3.2	2.9	0.0	16.3	1.6	0.89	^∗∗∗^	^∗∗∗^
SRL/LRB	1.9	1.8	0.0	7.3	0.6	0.77	^∗∗∗^	^∗∗∗^	3.0	2.9	0.8	7.4	0.8	0.87	^∗∗∗^	^∗∗∗^	3.3	3.2	0.0	19.4	1.1	0.87	^∗∗∗^	^∗∗∗^
SRL/PRL	1.7	1.7	0.2	4.7	0.6	0.83	^∗∗∗^	^∗∗∗^	1.7	1.6	0.3	4.1	0.5	0.81	^∗∗∗^	^∗∗∗^	2.1	2.0	0.2	5.9	0.8	0.64	^∗∗∗^	^∗∗∗^
MED	1.0	1.0	1.0	3.0	0.1	0.88	^∗∗∗^	^∗∗∗^	1.6	1.0	1.0	19.0	1.6	0.85	^∗∗∗^	^∗∗∗^	2.7	1.0	1.0	29.0	2.7	0.80	^∗∗∗^	^∗∗∗^
MAX	6.3	6.0	1.0	27.0	4.2	0.91	^∗∗∗^	^∗∗∗^	14.8	14.0	1.0	40.0	6.0	0.91	^∗∗∗^	^∗∗∗^	18.9	18.0	1.0	50.0	6.9	0.91	^∗∗∗^	^∗∗∗^
LED	1.7	1.6	0.0	6.7	0.9	0.86	^∗∗∗^	^∗∗∗^	2.6	2.4	0.0	7.9	1.4	0.84	^∗∗∗^	^∗∗∗^	2.6	2.3	0.0	10.4	1.7	0.80	^∗∗∗^	^∗∗∗^
PER	107.8	103.9	0.5	359.2	56.5	0.91	^∗∗∗^	^∗∗∗^	322.0	310.8	0.6	851.1	122.5	0.91	^∗∗∗^	^∗∗∗^	471.5	460.6	1.1	1022.8	144.3	0.92	^∗∗∗^	^∗∗∗^
DIA	0.2	0.2	0.0	0.4	0.0	0.93	^∗∗∗^	^∗∗∗^	0.2	0.2	0.0	0.3	0.0	0.91	^∗∗∗^	^∗∗∗^	0.2	0.2	0.0	0.4	0.0	0.87	^∗∗∗^	^∗∗∗^
VOL	85.8	73.8	0.1	541.1	57.6	0.92	^∗∗∗^	^∗∗∗^	152.3	135.8	0.1	707.2	76.7	0.92	^∗∗∗^	^∗∗∗^	200.8	166.7	1.2	1141.5	127.4	0.88	^∗∗∗^	^∗∗∗^
PRA	11.2	10.9	0.0	34.5	4.1	0.93	^∗∗∗^	^∗∗∗^	19.4	19.2	2.1	37.7	4.9	0.91	^∗∗∗^	^∗∗∗^	25.8	24.9	0.6	68.8	8.4	0.89	^∗∗∗^	^∗∗∗^
TRLUpper	42.3	38.2	0.0	175.7	29.2	0.90	^∗∗∗^	^∗∗∗^	141.8	136.0	0.0	438.1	68.0	0.90	^∗∗∗^	^∗∗∗^	213.5	209.6	0.0	676.3	93.6	0.89	^∗∗∗^	^∗∗∗^
TRLLower	24.6	20.9	0.0	122.1	14.9	0.81	^∗∗∗^	^∗∗∗^	70.5	56.3	0.0	394.3	48.0	0.74	^∗∗∗^	^∗∗∗^	119.8	92.8	0.0	1701.8	90.5	0.75	^∗∗∗^	^∗∗∗^
CVA	65.3	56.3	0.0	360.1	48.0	0.88	^∗∗∗^	^∗∗∗^	287.4	268.8	0.0	963.3	142.8	0.90	^∗∗∗^	^∗∗∗^	489.3	490.5	0.0	1161.7	187.2	0.92	^∗∗∗^	^∗∗∗^
DEP	19.0	19.4	0.2	37.4	5.1	0.88	^∗∗∗^	^∗∗∗^	31.8	32.5	0.3	49.1	7.4	0.79	^∗∗∗^	^∗∗∗^	40.0	41.5	0.0	55.9	8.5	0.72	^∗∗∗^	^∗∗∗^
WID	6.0	5.4	0.1	22.4	3.3	0.88	^∗∗∗^	^∗∗∗^	16.1	15.9	0.1	33.8	5.7	0.90	^∗∗∗^	^∗∗∗^	21.1	21.6	0.3	34.3	5.8	0.92	^∗∗∗^	^∗∗∗^
WDR	0.3	0.3	0.0	2.8	0.2	0.77	^∗∗∗^	^∗∗∗^	0.5	0.5	0.0	2.3	0.2	0.76	^∗∗∗^	^∗∗∗^	0.6	0.5	0.0	52.0	0.8	0.08	0.084	0.053
NWA	0.7	0.6	0.0	2.4	0.4	0.91	^∗∗∗^	^∗∗∗^	2.3	2.2	0.0	7.5	1.0	0.92	^∗∗∗^	^∗∗∗^	3.7	3.6	0.0	20.6	1.5	0.91	^∗∗∗^	^∗∗∗^
RHZO	1145	1080	7.5	3792	651	0.91	^∗∗∗^	^∗∗∗^	3696	3589	9.3	9783	1562	0.92	^∗∗∗^	^∗∗∗^	5680	5589	14.0	14305	1991	0.92	^∗∗∗^	^∗∗∗^
LRB	37.3	36.0	0.0	108.0	19.7	0.87	^∗∗∗^	^∗∗∗^	70.8	70.0	0.0	330.0	24.7	0.85	^∗∗∗^	^∗∗∗^	104.4	100.0	0.0	238.0	39.3	0.84	^∗∗∗^	^∗∗∗^
NLR	46.9	46.0	1.0	241.0	25.0	0.85	^∗∗∗^	^∗∗∗^	90.6	83.0	1.0	289.0	42.0	0.81	^∗∗∗^	^∗∗∗^	149.6	137.0	0.0	507.0	68.7	0.70	^∗∗∗^	^∗∗∗^
IRB	46.8	46.0	2.0	201.0	23.2	0.87	^∗∗∗^	^∗∗∗^	86.4	82.0	2.0	280.0	35.3	0.83	^∗∗∗^	^∗∗∗^	139.2	130.0	2.0	424.0	59.2	0.73	^∗∗∗^	^∗∗∗^
TRArea	6.2	5.6	0.0	27.5	3.6	0.92	^∗∗∗^	^∗∗∗^	18.7	17.7	0.0	59.0	8.3	0.92	^∗∗∗^	^∗∗∗^	28.1	27.1	0.0	122.7	11.3	0.91	^∗∗∗^	^∗∗∗^
TRAUpper	3.8	3.2	0.0	21.9	2.7	0.92	^∗∗∗^	^∗∗∗^	12.4	11.8	0.0	41.9	6.1	0.91	^∗∗∗^	^∗∗∗^	17.8	17.1	0.0	64.9	8.7	0.90	^∗∗∗^	^∗∗∗^
TRALower	2.2	2.0	0.0	10.3	1.1	0.83	^∗∗∗^	^∗∗∗^	6.0	4.6	0.0	42.8	4.1	0.68	^∗∗∗^	^∗∗∗^	9.6	7.7	0.0	112.6	6.6	0.75	^∗∗∗^	^∗∗∗^
SOL	0.0	0.0	0.0	0.3	0.0	0.78	^∗∗∗^	^∗∗∗^	0.0	0.0	0.0	0.4	0.0	0.10	^∗∗∗^	^∗∗∗^	0.0	0.0	0.0	0.7	0.0	0.04	^∗∗∗^	^∗∗^
BSH	5.9	5.0	1.0	26.0	3.9	0.86	^∗∗∗^	^∗∗∗^	10.7	10.0	1.0	35.0	5.8	0.75	^∗∗∗^	^∗∗∗^	10.5	9.0	1.0	50.0	7.0	0.61	^∗∗∗^	^∗∗∗^
COM	0.2	0.2	0.0	1.0	0.1	0.54	^∗∗∗^	^∗∗∗^	0.3	0.3	0.1	1.0	0.1	0.42	^∗∗∗^	^∗∗∗^	0.3	0.3	0.1	1.0	0.1	0.40	^∗∗∗^	^∗∗∗^
CMT	0.2	0.2	0.0	0.3	0.0	0.48	^∗∗∗^	^∗∗∗^	0.2	0.2	0.0	0.3	0.0	0.26	^∗∗∗^	^∗∗∗^	0.2	0.2	0.0	0.3	0.1	—	^∗∗∗^	^∗∗∗^
CMM	0.4	0.4	0.0	0.7	0.1	0.61	^∗∗∗^	^∗∗∗^	0.4	0.4	0.0	0.7	0.1	0.46	^∗∗∗^	^∗∗∗^	0.4	0.4	0.0	0.7	0.1	0.26	^∗∗∗^	^∗∗∗^
CMB	0.9	1.0	0.7	1.0	0.1	0.29	^∗∗∗^	^∗∗∗^	0.9	1.0	0.0	1.0	0.1	0.46	^∗∗∗^	^∗∗∗^	0.8	0.8	0.0	1.0	0.1	0.27	^∗∗∗^	^∗∗∗^
COP	0.4	0.4	0.2	1.0	0.1	0.42	^∗∗∗^	^∗∗∗^	0.4	0.4	0.1	1.0	0.1	0.57	^∗∗∗^	^∗∗∗^	0.5	0.5	0.1	1.0	0.1	0.50	^∗∗∗^	^∗∗∗^
CPT	0.2	0.2	0.0	0.3	0.0	0.50	^∗∗∗^	^∗∗∗^	0.2	0.2	0.0	0.3	0.0	—	^∗∗^	^∗∗∗^	0.2	0.2	0.0	0.3	0.0	0.24	^∗∗∗^	^∗∗∗^
CPM	0.4	0.4	0.0	0.7	0.1	0.66	^∗∗∗^	^∗∗∗^	0.4	0.4	0.0	0.7	0.1	0.66	^∗∗∗^	^∗∗∗^	0.5	0.5	0.0	0.7	0.1	0.50	^∗∗∗^	^∗∗∗^
CPB	1.0	1.0	0.7	1.0	0.1	0.26	^∗∗∗^	^∗∗∗^	0.9	1.0	0.0	1.0	0.1	0.49	^∗∗∗^	^∗∗∗^	0.9	0.8	0.0	1.0	0.1	0.29	^∗∗∗^	^∗∗∗^
LRA	85.4	90.0	2.0	90.0	15.5	0.31	^∗∗∗^	^∗∗∗^	86.1	90.0	2.0	90.0	10.8	0.45	^∗∗∗^	^∗∗∗^	88.3	90.0	2.0	90.0	9.0	0.34	^∗∗∗^	^∗∗∗^

^∗^<0.055; ^∗∗^<0.01; ^∗∗∗^<0.001. SB = subblock. RSA trait name descriptions and units are found in Table 1.

**Table 3:** Five iRoot categories. Mean root trait values of the top 10 ranked (of 292 total) genotypes for each iRoot category at 9 days after germination. Only italic values were used to calculate iRoot rankings.

iRoot category	TRL	PRL	WID	CVA	LRB	VOL	LRA	SOL2	LED	RHZO	TRL_GR	TRLUpper	Root_weight
N. Foraging	288	37.7	*20.2* ^+^	393	79	201	87.7	129	3.46	4841	*201* ^+^	*211* ^+^	0.041
Drought Tol.	266	*37.3* ^+^	17.9	341	80	166	*85.8* ^−^	*121* ^−^	2.73	4513	*188* ^+^	176	0.038
Beard	*225* ^+^	37.2	*16.1* ^−^	304	*81* ^+^	153	*85.8* ^−^	*129* ^−^	*2.3* ^−^	3852	152	140	0.038
Umbrella	249	*38.5* ^+^	*19.5* ^+^	*380* ^+^	77	207	*88.3* ^+^	145	*3.5* ^+^	4250	171	183	0.044
Maximum	*289* ^+^	*38.1* ^+^	*19.8* ^+^	*386* ^+^	*82* ^+^	*211* ^+^	87.7	127	3.13	*4826* ^+^	201	205	*0.043* ^+^

Italic values denote root trait used in iRoot category. ^+^Higher value of the trait is favorable for the specific iRoot category. ^−^Lower value of the trait is favorable for the specific iRoot category.

**Table 4:** Summary data for genotype-, phenotype-, and shape-based clusters. Summary in respect to country of origin, maturity group (MG), growth habit, diversity, root traits (TRL, PRL, WID, CVA, LRB, VOL, LRA, SOL2, LED, RHZO, TRL_GR, TRLUpper, and Root_weight) at 9 days after germination, iRoot category rankings, and phenotype-based cluster mean value. iRoot mean rank is calculated from the scores of all the genotypes within the specific GBC, PBC, or shape-based cluster. PBC mean was calculated by averaging the PBC groupings of genotypes that comprised the cluster.

	Genotype-based cluster								Phenotype-based cluster								Shape-based cluster							
Country	A	B	C	D	E	F	G	H	A	B	C	D	E	F	G	H	A	B	C	D	E	F	G	H
China	3	48	14	37	4	2	31	19	1	15	54	27	46	6	8	1	20	24	24	25	14	14	18	19
Japan	0	8	3	1	27	5	2	0	1	3	9	9	16	3	3	2	5	4	6	4	3	5	14	5
Korea	3	17	1	0	7	0	3	0	1	0	17	5	7	1	0	0	5	5	4	5	4	1	2	5
Other	1	6	2	3	2	1	2	0	0	1	5	4	4	1	2	0	2	1	2	2	2	1	4	3
Russia	0	16	0	0	0	0	0	1	0	4	7	3	2	0	0	1	0	0	7	0	3	3	1	3
USA	22	1	0	0	0	0	0	0	0	0	6	8	8	1	0	0	3	1	5	6	2	5	1	0
	29	96	20	41	40	8	38	20	3	23	98	56	83	12	13	4	35	35	48	42	28	29	40	35

MG	A	B	C	D	E	F	G	H	A	B	C	D	E	F	G	H	A	B	C	D	E	F	G	H
1	1	12	0	3	2	0	0	1	0	2	8	4	4	1	0	0	5	4	2	3	2	2	0	1
2	7	45	12	22	2	6	17	4	1	12	32	28	27	5	6	4	12	11	18	19	11	10	17	17
3	20	39	8	16	36	2	21	14	2	8	58	23	52	6	7	0	18	20	15	26	28	15	12	22
4	1	0	0	0	0	0	0	1	0	1	0	1	0	0	0	0	0	0	0	0	1	1	0	0

Growth habit	A	B	C	D	E	F	G	H	A	B	C	D	E	F	G	H	A	B	C	D	E	F	G	H
Determinate	1	29	3	12	34	1	1	7	2	11	24	12	30	4	5	0	16	7	11	13	10	8	9	14
Semideterminate	2	7	4	18	3	0	3	3	0	2	16	7	12	2	0	1	2	0	5	7	4	6	1	9
Indeterminate	26	60	13	11	3	7	34	10	1	10	58	37	41	6	8	3	17	22	19	28	28	14	19	17

Diversity	A	B	C	D	E	F	G	H	A	B	C	D	E	F	G	H	A	B	C	D	E	F	G	H
Diverse	9	1	0	0	0	0	0	0	0	0	3	4	3	0	0	0	0	2	0	2	3	2	1	0
Elite	13	0	0	0	0	0	0	0	0	0	3	4	5	1	0	0	0	1	1	3	3	0	4	1
Landrace	7	95	20	41	40	8	38	20	3	23	92	48	75	11	13	4	35	32	34	43	36	26	24	39

Root trait	A	B	C	D	E	F	G	H	A	B	C	D	E	F	G	H	A	B	C	D	E	F	G	H
TRL	218	229	235	222	220	148	205	229	309	274	244	219	198	175	152	119	255	232	225	234	207	223	190	197
PRL	37.3	36.5	37.2	34.4	35.9	33.8	35.1	36.2	37.9	37.2	36.8	36.7	35.1	34.4	32.5	30.7	36.2	36.5	36.0	36.1	36.1	35.6	35.9	35.2
WID	16.7	17.1	17.8	15.3	16.4	12.0	15.9	16.9	20.6	19.2	17.7	16.6	15.2	13.5	12.6	9.8	18.9	17.3	16.8	17.0	16.2	16.6	14.5	14.2
CVA	316	319	336	272	300	207	283	312	406	368	333	308	273	240	208	155	354	321	310	313	298	302	267	254
LRB	72.4	73.6	75.4	75.2	73.3	59.5	67.5	72.6	78.9	78.7	75.5	73.9	70.2	66.0	59.4	50.7	74.1	74.0	72.3	75.7	71.1	70.5	68.9	71.8
VOL	163	168	182	154	165	96.1	129	164	209	189	171	164	146	137	107	87.7	180	167	162	166	155	149	145	143
LRA	87.1	87.0	87.5	86.6	87.0	85.9	86.5	87.0	87.5	87.2	87.2	87.1	86.6	86.4	86.1	86.4	87.2	87.4	86.7	86.8	86.8	86.9	86.9	86.7
SOL2	138	133	136	116	130	133	132	130	125	127	129	134	131	131	131	126	132	132	131	127	137	129	134	123
LED	2.77	2.81	3.01	2.33	2.77	1.95	2.63	2.65	3.13	2.98	2.86	2.72	2.57	2.22	2.10	1.93	2.98	2.89	2.80	2.71	2.76	2.61	2.53	2.24
RHZO	3754	3914	4033	3756	3712	2620	3566	3926	5279	4654	4177	3753	3387	3004	2685	2073	4361	3959	3848	3996	3544	3815	3244	3383
TRL_GR	148	157	162	149	148	99.4	136	157	213	185	166	150	134	113	104	82.9	172	159	152	160	142	150	128	131
TRLUpper	149	156	165	140	150	87.1	136	152	220	189	166	148	130	109	91.5	72.0	176	159	152	156	141	147	126	123
Root_weight	0.102	0.100	0.099	0.091	0.101	0.047	0.072	0.085	0.144	0.111	0.101	0.097	0.086	0.073	0.053	0.045	0.108	0.099	0.096	0.098	0.090	0.093	0.081	0.078
PBC mean	4.00	3.72	3.35	4.15	4.12	6.88	4.55	3.70	1.00	2.00	3.00	4.00	5.00	6.00	7.00	8.00	2.71	3.6	3.89	4.52	4.52	3.93	5.24	4.97

iRoot mean rank	A	B	C	D	E	F	G	H	A	B	C	D	E	F	G	H	A	B	C	D	E	F	G	H
Drought Tol.	158	134	135	157	150	239	182	132	5	30	85	140	217	255	282	291	116	142	146	108	182	136	191	160
N. Foraging	149	119	90	171	155	282	188	132	22	57	95	150	203	219	247	247	57	108	135	116	175	146	212	228
Umbrella	117	119	77	202	152	263	189	137	3	20	80	151	217	265	280	291	80	104	136	138	155	155	180	222
Beard	184	157	189	68	143	132	162	145	25	60	95	137	202	244	273	275	172	182	160	118	177	138	156	83
Maximum	128	116	87	170	151	277	212	143	141	130	143	159	151	138	132	133	71	113	138	122	171	154	197	211
iRoot rank mean	147	129	116	154	150	239	187	138	39	59	100	147	198	224	243	247	99	130	143	120	172	146	187	181

**Table 5:** Mean fixation indices. Indices based on SNP value comparison between genotype-based clusters (GBC) (low number = low diversity, high number = high diversity).

		A	B	C	D	E	F	G
Genotype-based clusters	B	0.123						
	C	0.202	0.165					
	D	0.199	0.112	0.243				
	E	0.244	0.146	0.308	0.156			
	F	0.339	0.297	0.370	0.286	0.391		
	G	0.298	0.260	0.292	0.251	0.380	0.245	
	H	0.195	0.118	0.233	0.105	0.218	0.259	0.142

## Data Availability

The ARIA 2.0 code is freely available at the address: https://bitbucket.org/baskargroup/aria2/src/master/. Analysis code is freely available at the address: https://github.com/mighster/ARIA2.0. Root extracted data, raw images and/or segmented masks are available upon request.

## Supplementary Materials

Supplementary 1 Supplementary Figure S1: Bayes inflection curve based on genotypic values. Supplementary Figure S2: mean root shape outline. Shapes were generated from all the root images for a genotype using via Elliptical Fourier Transformation (EFT). Supplementary Figure S3: architecture of the deep convolution autoencoder model. This model was used to represent the reconstructed mean root shape profile image in an eight-dimensional (high-level feature) vector. Supplementary Figure S4: Pearson's correlations of 51 root traits at (a) 6 and (b) 12 days after germination. Root traits measured on 292 soybean accession (replications = 14). Hierarchical clustering was used to group similar traits. Symbols (shape and color) denote RSA traits used in the corresponding iRoot index (cumulative trait scores). Supplementary Figure S5: segmented binary images of PI 578367, PI 89134, and PI 507491. Supplementary Figure S6: dendrogram displaying genomic and country of origin relationships of 292 soybean genotypes. Eight genotype-based clusters (GBC) based on genetic distances are displayed as the tree's branch colors. Genotype's country of origin is displayed as the tree's leaf text and colors. Supplementary Figure S7: correlations between phenotypes (*x*-axis) and genotypes (*y*-axis). Supplementary Figure S8: eight genotype-based cluster performance based on 13 root traits. Supplementary Figure S9: nine RSA traits displaying the increase in broad-sense heritability (*H*^2^) with each replicate tested (*n* = 14).

Supplementary 2 Supplementary Table S1: Tukey's Honest Significant Difference comparisons among the three diversity groups. Supplementary Table S2: variance inflation factor results show high collinearity between root traits. Supplementary Table S3: alternate table to Table 4 summarizing soybean root values and metadata. Supplementary Table S4: Tukey's Honest Significant Difference groupings for the 13 traits used to develop iRoot categories. Supplementary Table S5: presence of the top 25 ranked iRoot from each category within the genotype-based cluster.
