# Supplementary material for: Soybean Root System Architecture Trait Study through Genotypic, Phenotypic, and Shape-Based Clusters
Source: Plant Phenomics. 2020 Jun 9;2020:1925495. doi: 10.34133/2020/1925495 (PMC7706349; doi:10.34133/2020/1925495)
Supplement: Supplementary 1 — Supplementary Figure S1: Bayes inflection curve based on genotypic values. Supplementary Figure S2: mean root shape outline. Shapes were generated from all the root images for a genotype using via Elliptical Fourier Transformation (EFT). Supplementary Figure S3: architecture of the deep convolution autoencoder model. This model was used to represent the reconstructed mean root shape profile image in an eight-dimensional (high-level feature) vector. Supplementary Figure S4: Pearson's correlations of 51 root traits at (a) 6 and (b) 12 days after germination. Root traits measured on 292 soybean accession (replications = 14). Hierarchical clustering was used to group similar traits. Symbols (shape and color) denote RSA traits used in the corresponding iRoot index (cumulative trait scores). Supplementary Figure S5: segmented binary images of PI 578367, PI 89134, and PI 507491. Supplementary Figure S6: dendrogram displaying genomic and country of origin relationships of 292 soybean genotypes. Eight genotype-based clusters (GBC) based on genetic distances are displayed as the tree's branch colors. Genotype's country of origin is displayed as the tree's leaf text and colors. Supplementary Figure S7: correlations between phenotypes (x-axis) and genotypes (y-axis). Supplementary Figure S8: eight genotype-based cluster performance based on 13 root traits. Supplementary Figure S9: nine RSA traits displaying the increase in broad-sense heritability (H2) with each replicate tested (n = 14). [file 1925495.f1.docx]

Title

*Full title:* Soybean root system architecture traits study through genotypic, phenotypic and shape-based clusters.

*Short title:* Cluster analysis of soybean root traits.

**Authors**

Authors: Kevin G. Falk^1^, Talukder Zaki Jubery^2^, Jamie A. O’Rourke^1,3^, Arti Singh^1^, Soumik Sarkar^2^, Baskar Ganapathysubramanian^2,*^, Asheesh K. Singh^1,*^


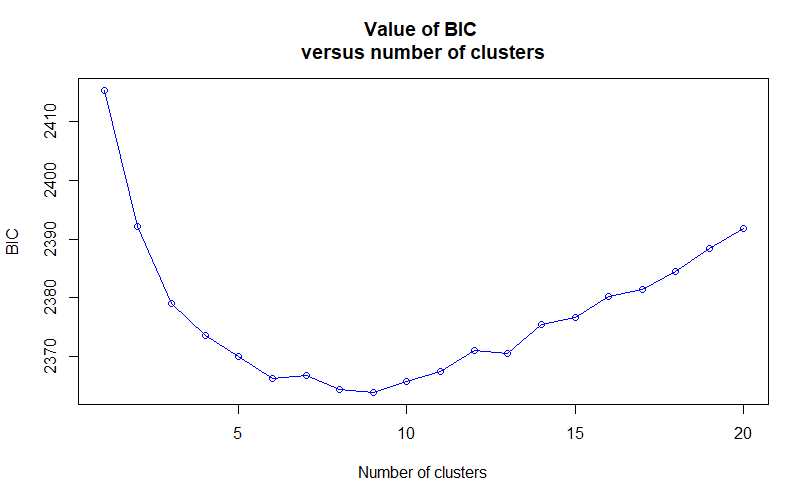


**Supplementary Figure S1:** Bayes inflection curve based on genotypic values.


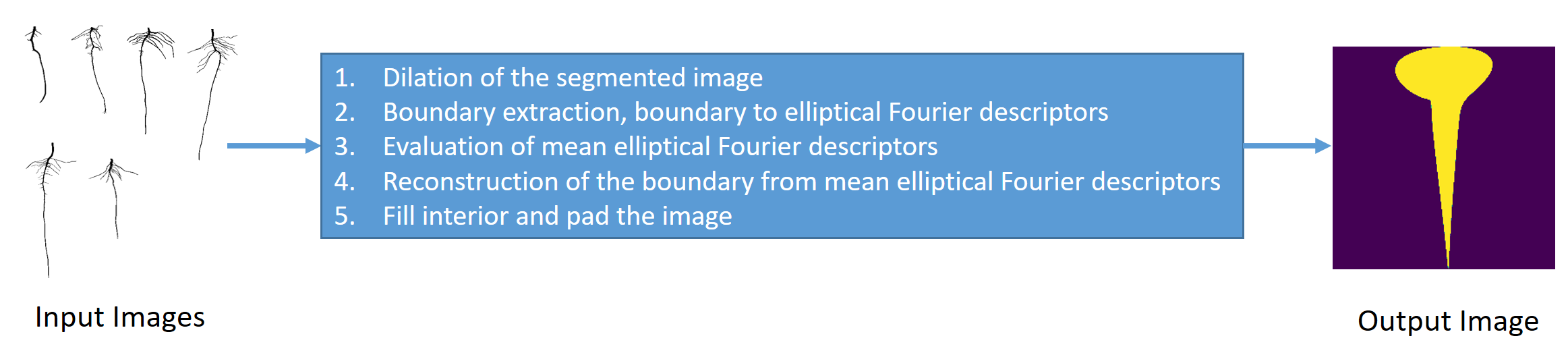


**Supplementary Figure S2:** Mean root shape outline. Shapes were generated from all the root images for a genotype using via Elliptical Fourier Transformation (EFT)


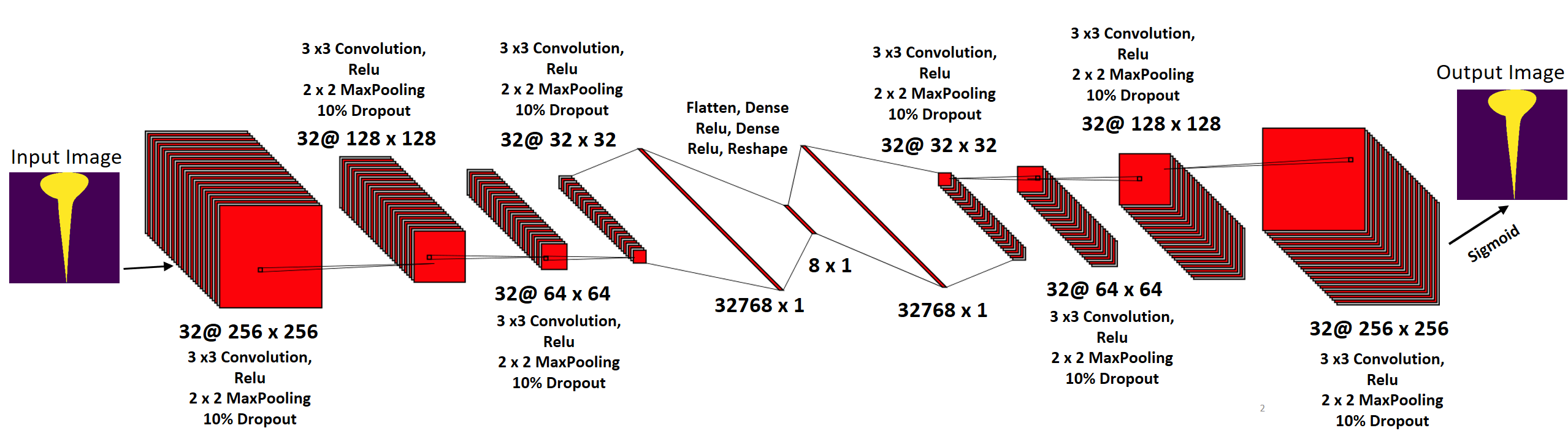


**Supplementary Figure S3:** Architecture of the deep convolution autoencoder model. This model was used to represent the reconstructed mean root shape profile image in an eight dimensional (high-level feature) vector.


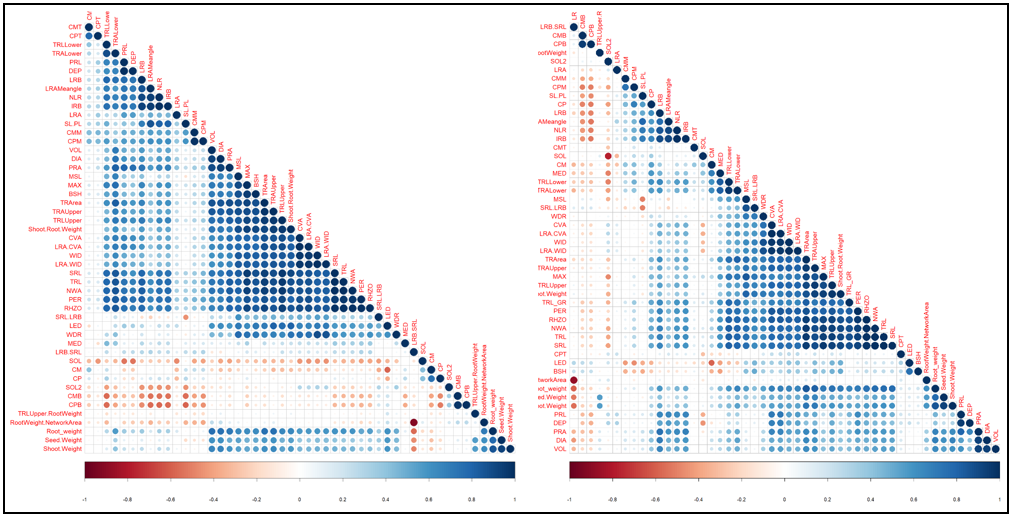


**Supplementary Figure S4:** Pearson correlations of 51 root traits at (a) 6 and (b) 12 days after germination. Root traits measured on 292 soybean accession (replications = 14). Hierarchical clustering was used to group similar traits. Symbols (shape and color) denote RSA traits used in corresponding iRoot index (cumulative trait scores).


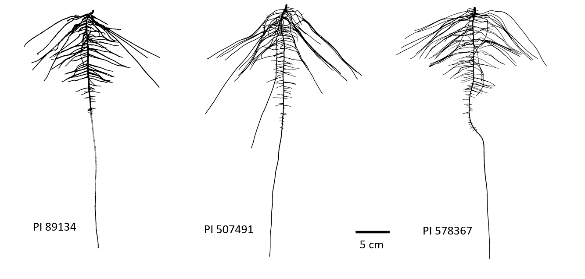


**Supplementary Figure S5:** Segmented binary images of PI 578367, PI 89134 and PI 507491.


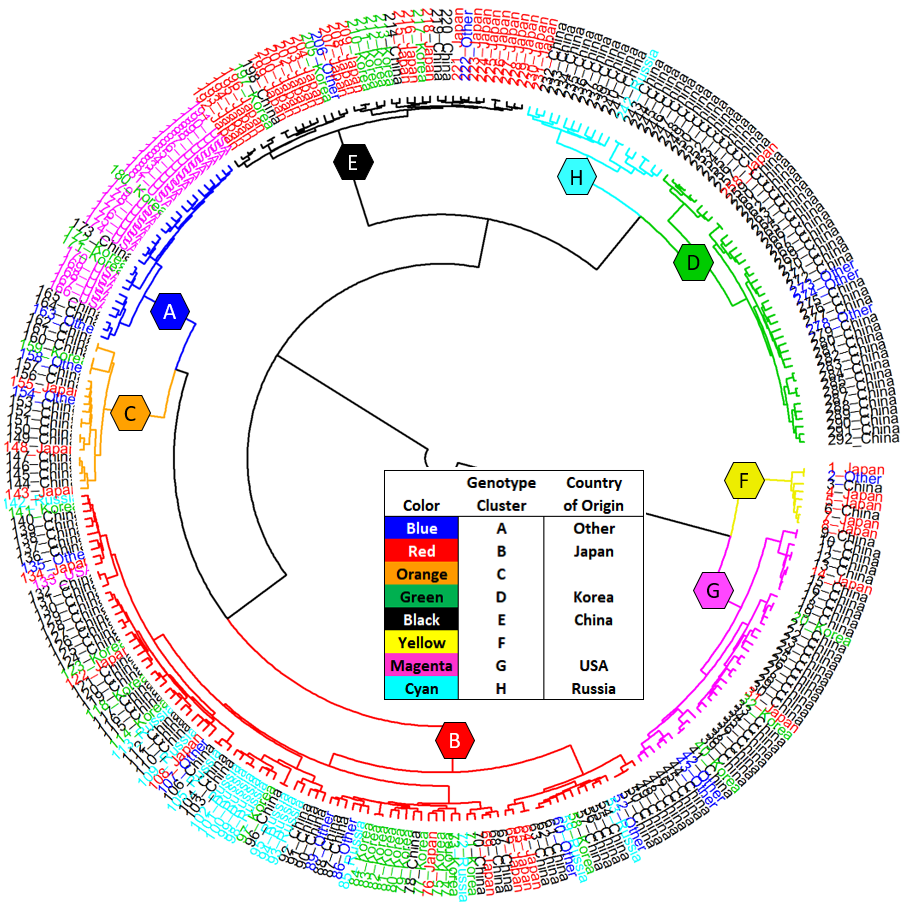


**Supplementary Figure S6:** Dendrogram displaying genomic and country of origin relationships of 292 soybean genotypes. Eight genotype-based clusters (GBC) based on genetic distances are displayed as the tree’s branch colors. Genotype’s country of origin are displayed as the tree’s leaf text and colors.


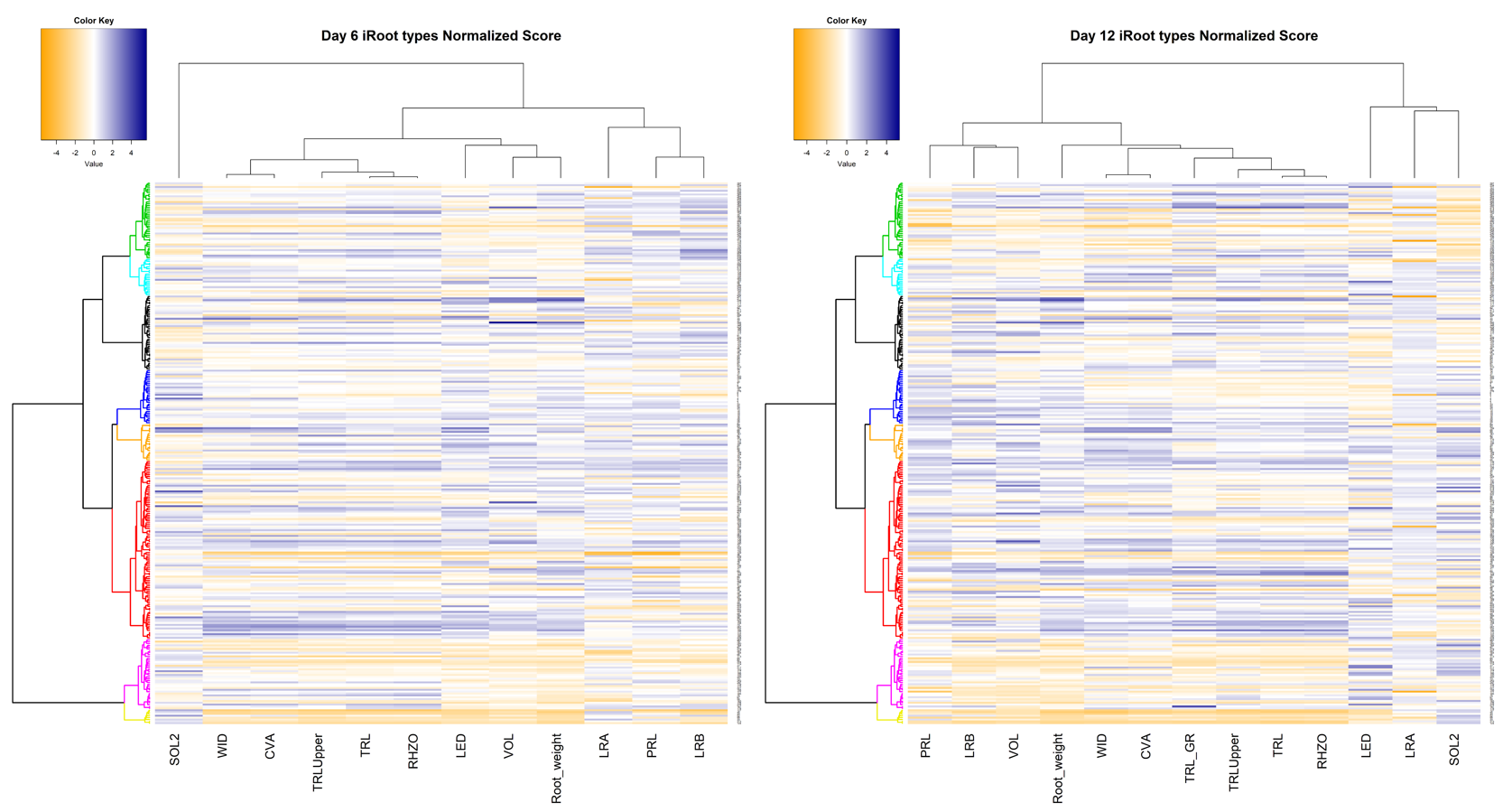


**Supplementary Figure S7:** Correlations between phenotypes (x-axis) and genotypes (y-axis). Dendrogram on the x-axis developed using SNP-based clusters, y-axis displays 13 RSA traits (TRL, PRL, WID, CVA, LRB, VOL, LRA, SOL2, LED, RHZO, TRL_GR, TRLUpper, Root weight) at (a) 6d (b) 12d.

**
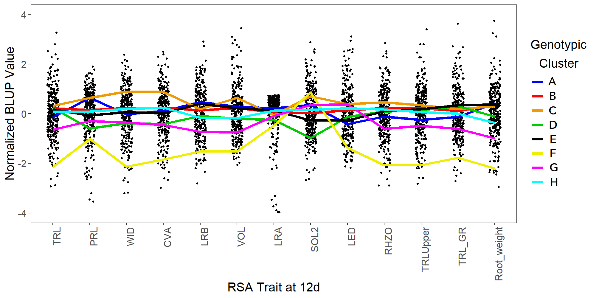

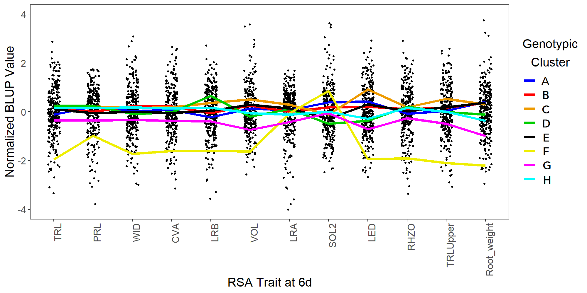
**

**Supplementary Figure S8:** Eight genotype-based clusters performance based on 13 root traits. Traits depicted include TRL, PRL, WID, CVA, LRB, VOL, LRA, SOL2, LED, RHZO, TRL_GR, TRLUpper, Root weight at 6 and 12 days after germination.


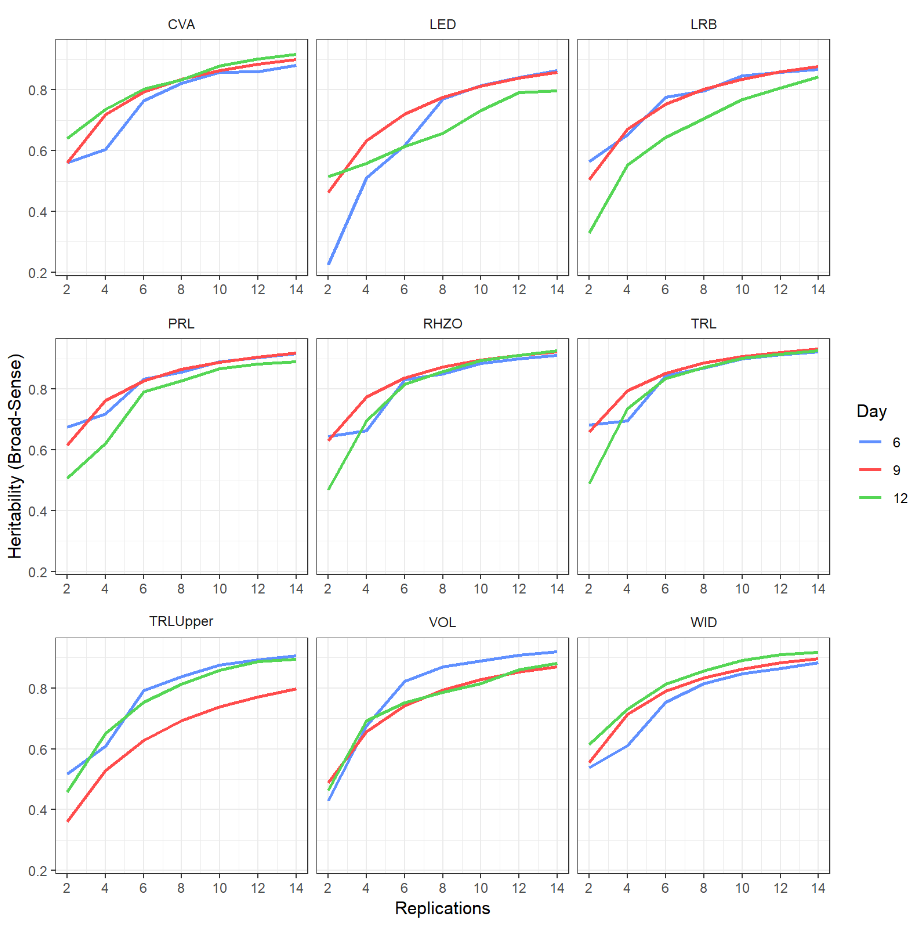


**Supplementary Figure S9:** Nine RSA traits displaying the increase in broad-sense heritability (H^2^) with each replicate tested (n=14).
