# Supplementary material for: Soybean Root System Architecture Trait Study through Genotypic, Phenotypic, and Shape-Based Clusters
Source: Plant Phenomics. 2020 Jun 9;2020:1925495. doi: 10.34133/2020/1925495 (PMC7706349; doi:10.34133/2020/1925495)

Title

*Full title:* Soybean root system architecture traits study through genotypic, phenotypic and shape-based clusters.

*Short title:* Cluster analysis of soybean root traits.

**Authors**

Authors: Kevin G. Falk^1^, Talukder Zaki Jubery^2^, Jamie A. O’Rourke^1,3^, Arti Singh^1^, Soumik Sarkar^2^, Baskar Ganapathysubramanian^2,*^, Asheesh K. Singh^1,*^

**Supplementary Table S1: Tukey’s Honest Significant Difference comparisons among the three diversity groups.** These groups include elite, diverse, landrace from 292 genotypes for root system architecture traits at 6, 9 and 12 days after germination.


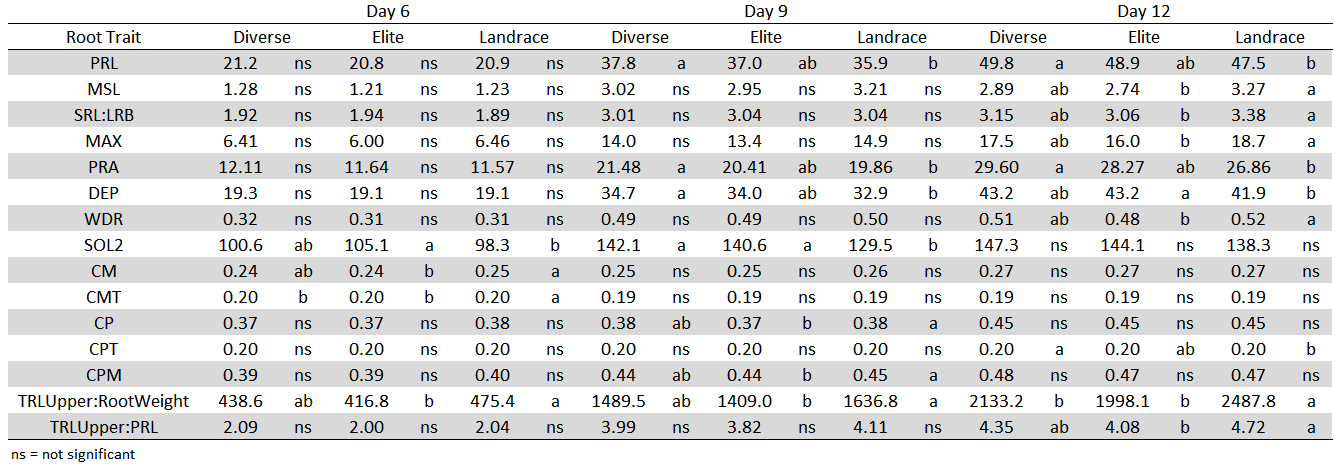


**Supplementary Table S2: Variance inflation factor results show high collinearity between root traits.**


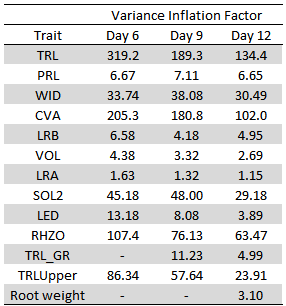


**Supplementary Table S3: Alternate table to Table 4 summarizing soybean root values and metadata.** Summary data included for genotype, phenotype and shape-based clusters in respect to country of origin, maturity group (MG), growth habit, diversity, root traits (TRL, PRL, WID, CVA, LRB, VOL, LRA, SOL2, LED, RHZO, TRL_GR, TRLUpper, Root weight) at 9 days after germination, iRoot category rankings and phenotype-based cluster mean value. Conditional formatting used to highlight trends; darker green color denotes high values with respect to the other 8 clusters.


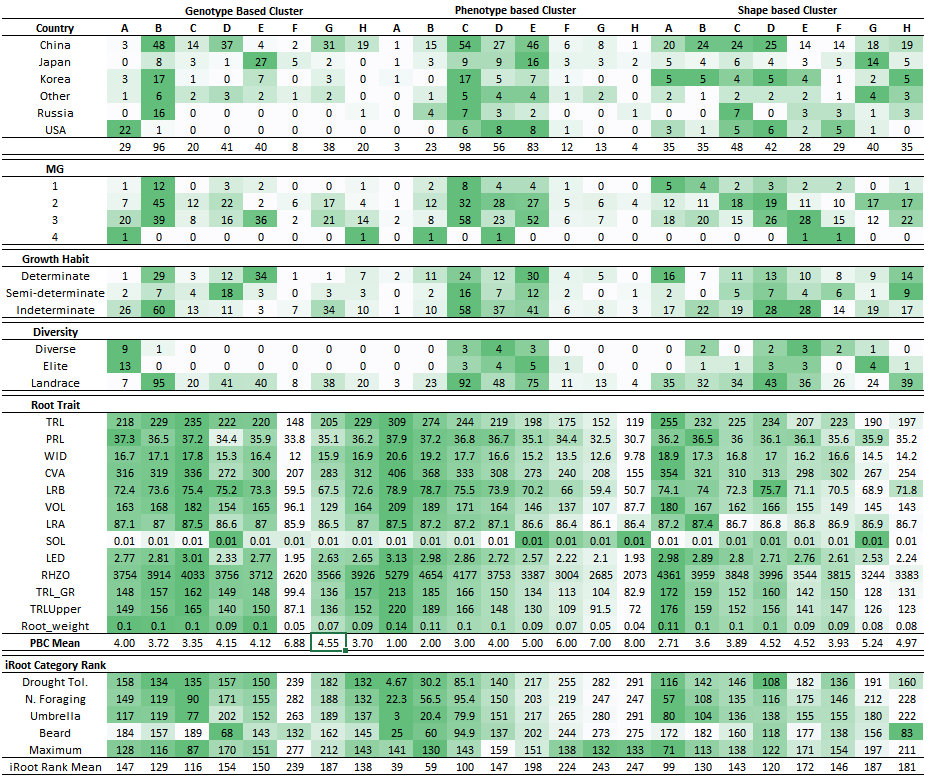


**Supplementary Table S4: Tukey’s Honest Significant Difference groupings for the 13 traits used to develop iRoot categories.**


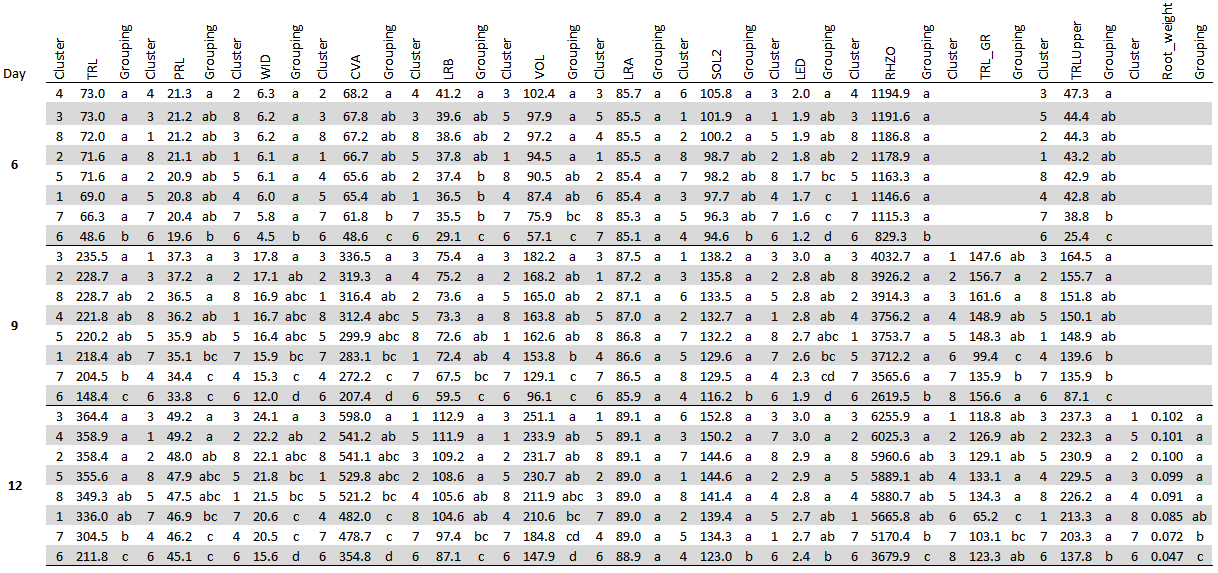


**Supplementary Table S5: Presence of the top 25 ranked iRoot from each category within the genotype-based cluster.** The number of individuals and the percent distribution with respect to the cluster size (n).


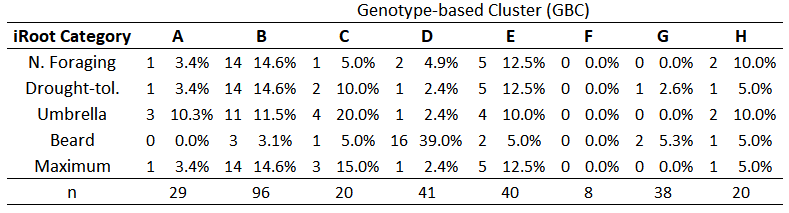

Supplement: Supplementary 2 — Supplementary Table S1: Tukey's Honest Significant Difference comparisons among the three diversity groups. Supplementary Table S2: variance inflation factor results show high collinearity between root traits. Supplementary Table S3: alternate table to Table 4 summarizing soybean root values and metadata. Supplementary Table S4: Tukey's Honest Significant Difference groupings for the 13 traits used to develop iRoot categories. Supplementary Table S5: presence of the top 25 ranked iRoot from each category within the genotype-based cluster. [file 1925495.f2.docx]
